# Directional asymmetry in gonad length indicates moray eels (Teleostei, Anguilliformes, Muraenidae) are “right-gonadal”

**DOI:** 10.1038/s41598-023-29218-3

**Published:** 2023-02-20

**Authors:** Yu-Jia Lin, Hong-Ming Chen

**Affiliations:** 1 Institute of Marine Ecology and Conservation, National Sun Yat-Sen University, Kaohsiung, Taiwan; 2 Department of Aquaculture, National Taiwan Ocean University, Keelung, Taiwan; 3 Center of Excellence for the Oceans, National Taiwan Ocean University, Keelung, Taiwan

**Keywords:** Ichthyology, Ecology, Evolution, Zoology

## Abstract

Directional asymmetry indicates a unidirectional deviation from perfect bilateral symmetry, which was rarely examined in the inner organs of the teleost (Teleostei) compared to external traits. This study examines the directional asymmetry in the gonad length of 20 species of moray eels (Muraenidae) and two outgroup species with 2959 individuals. We tested three hypotheses: (1) moray eel species did not exhibit directional asymmetry in the gonad length; (2) the directional asymmetry pattern was the same for all selected species; (3) the directional asymmetry was not related to the major habitat types, depth and size classes, and taxonomic closeness of the species. Moray eels were generally “right-gonadal”, the right gonad length being constantly and significantly longer than the left one in all studied Muraenidae species. The degree of asymmetry varied among species and was not significantly related to taxonomic closeness. The habitat types, depth, and size classes had intermingled effects on observed asymmetry without a clear correspondence. The directional asymmetry in the gonad length is a unique and widely occurring phenomenon in the Family Muraenidae, which was likely a by-product in the evolutionary history without significant disadvantage in survival.

## Introduction

Bilateral symmetry, the most common form of symmetry in the animal kingdom, involves reflection across an axis of symmetry (usually along the left–right axis)^1^. The deviation from perfect bilateral symmetry is called asymmetry and directional asymmetry indicates that such deviation is biased in one direction^2,3^. Directional asymmetry is commonly found in nature and taxonomically widespread^1,4^. Directional asymmetry can be an adaptation to selection^5^, a consequence due to stresses in the environments^6^, or simply an evolutionary by-product arising from mutations from pleiotropic genes^7,8^.

In teleosts (Teleostei), the asymmetry in the inner organs has received far less attention compared to external morphological traits^8,9^, with an exception for the otolith^10^. Some cases were reported of directional asymmetry in gonad sizes, such as the albacore *Thunnus alalunga*^11^, the pollan *Coregonus autumnalis*^12^, the ayu *Plecoglossus altivelis*^13^, and the whitefish *Coregonus* sp.^14^ and *Coregonus lavaretus*^15^. The limitation of these studies included limited sample sizes, small size ranges, and only single species analysis. Systematic studies with large sample sizes, wide size ranges, and multiple congeneric species examined simultaneously are still lacking.

Belonging to Anguilliformes, an elongated and eel-like Order with at least 16 families, the moray eels (Family: Muraenidae) is a large family with about 16 genera and 224 species^16^. In recent years, the taxonomy of moray eels has advanced greatly and scientists keep discovering new species in this family around the world^17–22^. Moray eels could reach 12 years old^23^ and three groups of sexuality were found: gonochoristic, protogynous hermaphrodite, and simultaneous hermaphrodite^24^. Moray eels are important components of coral reef ecosystems as top predators, feeding on fish and other large invertebrates^25^. Being carnivorous, and especially piscivorous, they play significant roles in regulating the abundance of numerous preys^26^, affecting the dynamics of coral reef-fish assemblages^27^, and controlling alien fish species such as the lionfish^28,29^. They are found to be able to communicate and coordinate with other fish species in hunting^30^.

Examination of the biology of the moray eels in Taiwan has continued for decades and we have accumulated a considerable amount of valuable data to examine the directional asymmetry of gonads with large sample sizes and numerous species examined simultaneously in a systematic manner. The major objective of this study is to examine the directional asymmetry in the gonad length of selected moray eel and outgroup species. Specifically, we are going to test the following hypothesis: (1) some moray eel species did not exhibit directional asymmetry in the gonad length; (2) the degree of directional asymmetry was the same for all examined species; (3) the degree of directional asymmetry was not related to the major habitat types, depth and size classes, and taxonomic closeness. We further propose hypotheses that (1) observed directional asymmetry in the gonad length did not affect reproduction success and (2) it is a neutral by-product of evolutionary history, rather than exogenous environmental factors without significant adaptive advantages.

## Results

A total of 22 species with 2,959 individuals and sufficient samples were selected for modeling directional asymmetry in gonad lengths. Among them 20 species from 5 genera belong to Muraenidae and two species, longfin snake-eel *Pisodonophis cancrivorus* (Ophichthidae) and shortbelly eel *Dysomma anguillare* (Synaphobranchidae) were included as the outgroup. The total lengths and weights by sex and species were summarized in Supplement material 1.

**Figure 1:**
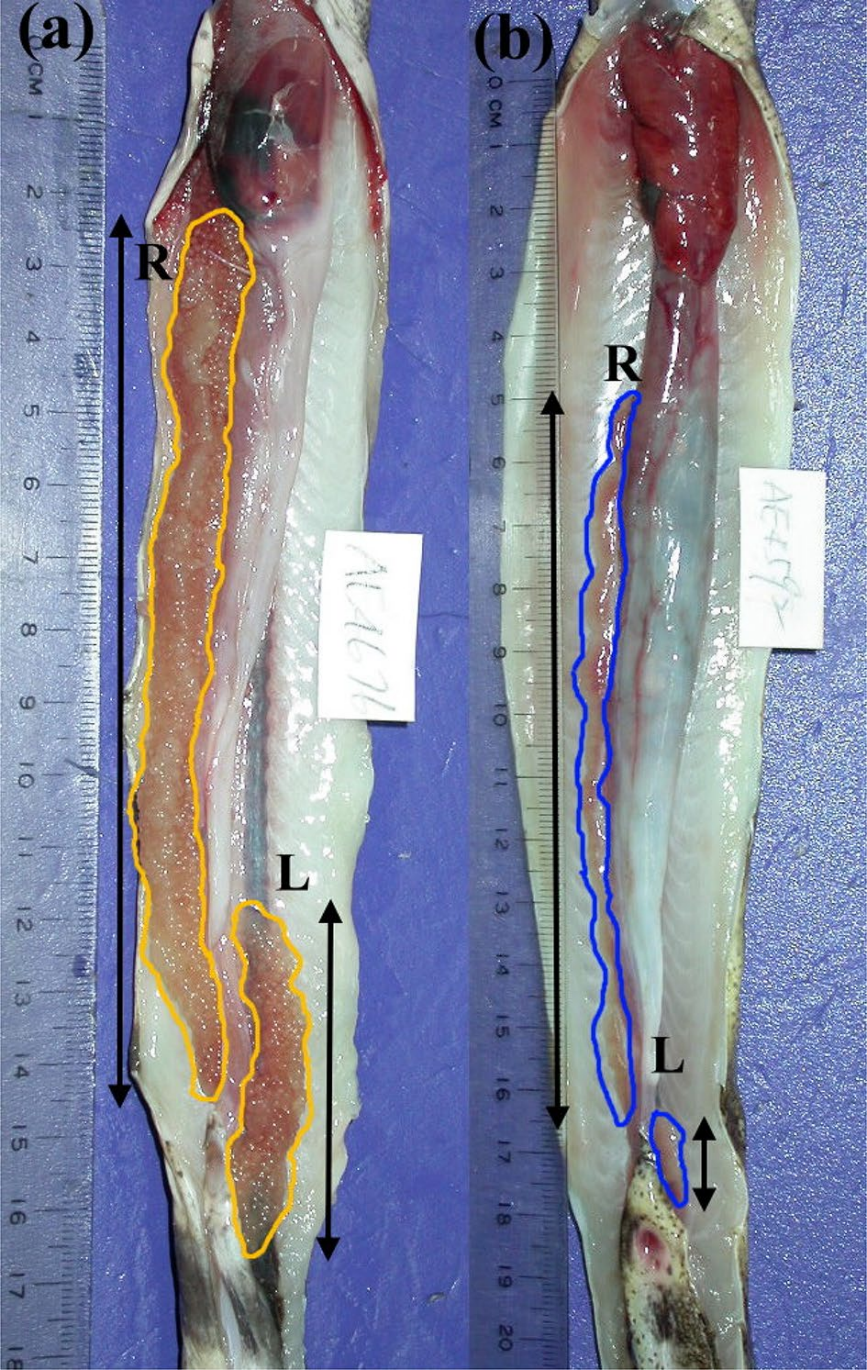
(**a**) A female *Gymnothorax minor*, collection ID: TOU-AE-4676 and (**b**) a male collection ID: TOU-AE-4592, showing the measurement of gonad length at both sides. The ovary is enclosed in orange and testis in blue. The left side (L) and right side (R) are defined when the fish is head up and belly down, and therefore, left–right direction appears opposite when the belly is up as shown this figure. Scale bar = 1 mm. (Photo credit: Huang, L.Y.)

The gonad length ratios were the highest in reticulated moray eel *Gymnothorax minor* (previously *Gymnothorax reticularis*) with a mean (± SD) of 3.45 ± 1.41 and 7.70 ± 4.73 for females and males, respectively (Fig. 1, Table 1). The natural log-transformed ratios were significantly larger than 0 (equivalent to a ratio larger than 1) in the studied Muraenidae species (all *p*s < 0.004), indicating the existence of directional asymmetry in gonad length. The Akaike Information Criterion (AIC) values for model selection are shown in Supplement material 2. For a majority (13 out of 20) of Muraenidae species, the males had stronger degrees of directional asymmetry in the gonad length than the females, as indicated by their effects (i.e., ln(GLR_M_–GLR_F_)) being significantly larger than 0 (all *p*s < 0.004, Table 1). The log-transformed gonad length ratios of two outgroup species were either close to zero (logged ratio = 0.051, *p* = 0.029 in *Pisodonophis cancrivorus*) or statistically zero (logged ratio = − 0.016, *p* = 0.474 in *Dysomma anguillare*, Table 1). The outgroup species had insignificant differences in gonad length ratio between sexes.

**Table 1:** Observed ratio of the right gonad length divided by the left (GLR), by species and sex (subscript F = female and set as the baseline and M = male), and estimated parameters (in natural logarithm, *p*-values in parenthesis) for the baseline gonad length ratio (GLR_F_), and the sexual difference (GLR_M_–GLR_F_).

Species	GLR_F_	GLR_M_	ln(GLR_F_)	ln(GLR_M_–GLR_F_)
*G. minor*	3.45 ± 1.41	7.70 ± 4.73	1.187 (< 0.001)	0.720 (< 0.001)
*G. chilospilus*	1.45 ± 0.28	1.57 ± 0.44	0.409 (< 0.001)	0
*G. eurostus*	1.21 ± 0.19	1.47 ± 0.46	0.176 (< 0.001)	0.167 (< 0.001)
*G. shaoi*	1.65 ± 0.34	2.30 ± 0.47	0.482 (< 0.001)	0.331 (< 0.001)
*G. prionodon*	1.98 ± 0.43	2.45 ± 0.80	0.666 (< 0.001)	0.188 (< 0.001)
*G. fimbriatus*	1.25 ± 0.21	1.46 ± 0.46	0.208 (< 0.001)	0.129 (0.002)
*G. flavimarginatus*	1.62 ± 0.36	2.32 ± 0.58	0.458 (< 0.001)	0.353 (< 0.001)
*G. hepaticus*	1.25 ± 0.19	1.42 ± 0.21	0.211 (< 0.001)	0.127 (0.001)
*G. kidako*	1.85 ± 0.45	2.49 ± 0.76	0.572 (< 0.001)	0.294 (< 0.001)
*G. pseudothyrsoideus*	1.34 ± 0.25	1.66 ± 0.31	0.273 (< 0.001)	0.211 (< 0.001)
*G. thyrsoideus*	1.42 ± 0.23	1.49 ± 0.27	0.363 (< 0.001)	0
*G. rueppelliae*	1.51 ± 0.30	1.95 ± 0.54	0.392 (< 0.001)	0.240 (0.001)
*G. neglectus*	1.99 ± 0.37	2.60 ± 2.59	0.723 (< 0.001)	0
*G. margaritophorus*	1.73 ± 0.47	2.42 ± 1.15	0.698 (< 0.001)	0
*G. pictus*	1.48 ± 0.14	1.42 ± 0.19	0.372 (< 0.001)	0
*E. polyzona*	1.11 ± 0.34	1.27 ± 0.89	0.114 (< 0.001)	0
*E. nebulosa*	1.75 ± 0.38	1.85 ± 0.40	0.565 (< 0.001)	0
*U. macrocephalus*	1.13 ± 0.19	1.44 ± 0.52	0.110 (0.078)	0.207 (0.004)
*U. micropterus*	1.26 ± 0.18	1.57 ± 0.35	0.220 (< 0.001)	0.206 (0.001)
*S. sathete*	1.88 ± 0.52	1.59 ± 0.35	0.604 (< 0.001)	-0.162 (0.004)
*P. cancrivorus*	1.06 ± 0.20	1.10 ± 0.10	0.051 (0.029)	0
*D. anguillare*	1.00 ± 0.10	0.96 ± 0.17	-0.016 (0.474)	0

Abbreviation for genera: *G*. *Gymnothorax*, *E*. *Echidna*, *U.* *Uropterygius*, *P*. *Pisodonophis*, *S*. *Strophidon* and *D*. *Dysomma.*

The studied species of Muraenidae had diverse relationships between the gonad length difference and both total length and sex (See Materials and Methods for details and Supplement material 3 for the demonstrating diagrams and Supplement 4 for AIC values of the candidate models for model selection). For example, the gonad length differences in *Gymnothorax minor* were significantly affected by the total length and sex (Supplement material 4). Moreover, the differences of *G. minor* females increased with total length faster than those of males (Table 2). This means that the degrees of directional asymmetry in the gonad length of *G. minor* became stronger as the size increased, particularly in females. On the other hand, the gonad length differences in *Gymnothorax thyrsoideus* were not affected by the total length and sex (Supplement material 4), with a difference significantly larger than zero (*p* < 0001, Table 2). Similar to the gonad length ratio, the gonad length differences of the two outgroup species were not affected significantly by the total length and sex. The gonad length differences of the two outgroup species were also either close to zero (0.644, *p* = 0.046 in *Pisodonophis cancrivorus*) or statistically zero (− 0.169, *p* = 0.544 in *Dysomma anguillare*, Table 2).

**Table 2:** Parameter estimates (A is the intercept and B is the slope) and corresponding *p*-values (in parentheses) for the best model representing the relationship between gonad length difference (GLD, right–left) and total length, and sex (subscript F and M for females and males, respectively).

Species	A_F_	B_F_	A_M_–A_F_	B_M_–B_F_
*G. minor*	− 1.570 (0.006)	0.250 (< 0.001)	1.859(0.032)	− 0.063 (0.001)
*G. chilospilus*	3.462 (< 0.001)	0.100 (< 0.001)	− 3.069 (< 0.001)	0
*G. eurostus*	0.223 (0.875)	0.041 (0.031)	0.805 (0.006)	0
*G. shaoi*	− 1.422 (0.097)	0.150 (< 0.001)	0.787 (0.004)	0
*G. prionodon*	− 0.661 (0.252)	0.140 (< 0.001)	0	0
*G. fimbriatus*	0.995 (0.210)	0.030 (0.042)	0	0.019 (0.008)
*G. flavimarginatus*	− 2.298 (< 0.001)	0.097 (< 0.001)	0.826 (0.013)	0
*G. hepaticus*	3.249 (0.093)	0.043 (0.216)	− 6.074 (0.024)	0.135 (0.005)
*G. kidako*	− 0.022 (0.981)	0.122 (< 0.001)	0	0
*G. pseudothyrsoideus*	3.821 (< 0.001)	0	0	0
*G. thyrsoideus*	3.297 (< 0.001)	0	0	0
*G. rueppelliae*	− 1.976 (0.118)	0.130 (< 0.001)	0	0
*G. neglectus*	− 0.539 (0.636)	0.158 (< 0.001)	0	− 0.041 (< 0.001)
*G. margaritophorus*	− 2.170 (0.204)	0.155 (< 0.001)	0	0
*G. pictus*	− 1.118 (0.208)	0.114 (< 0.001)	0	− 0.048 (< 0.001)
*E. polyzona*	1.143 (< 0.001)	0	0	0
*E. nebulosa*	0.526 (0.758)	0.108 (0.004)	0	0
*U. macrocephalus*	1.067 (< 0.001)	0	0	0
*U. micropterus*	− 1.040 (0.096)	0.106 (< 0.001)	0	0
*S. sathete*	8.077 (< 0.001)	0.033 (0.078)	− 6.784 (< 0.001)	0
*P. cancrivorus*	0.644 (0.046)	0	0	0
*D. anguillare*	− 0.169 (0.544)	0	0	0

**Figure 2:**
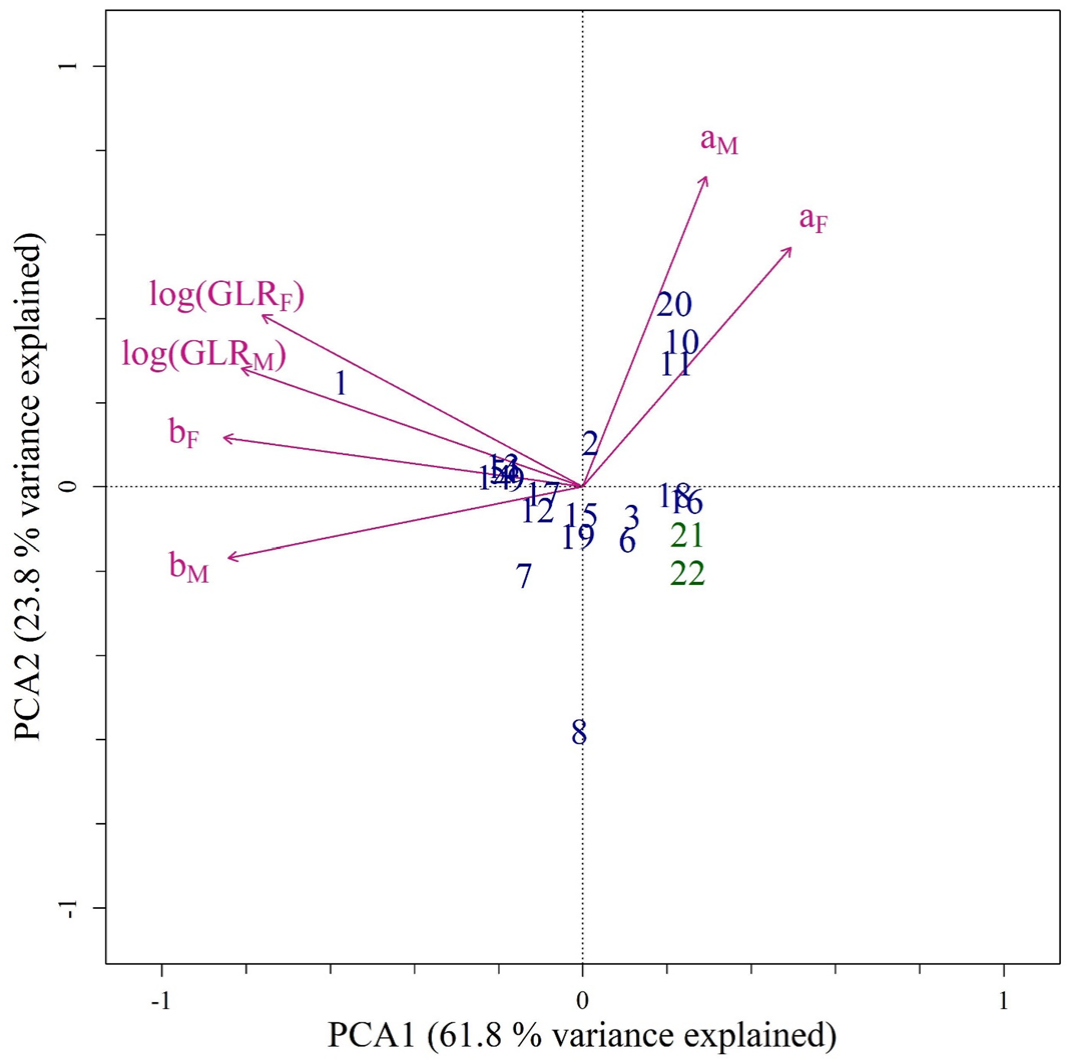
Biplot of the correlation-based principal component analysis on the standardized coefficients representing the directional symmetry in gonad length (log(GLR) is the natural log ratio of the gonad length, (**a**) and (**b**) is the intercept and slope of the relationship between the gonad length difference and the total length, respectively. Subscript F and M indicate females and males, respectively). The arrows indicate the correlation of variables to the first and second principal component axis (PCA1, 61.8%of total variance explained and PCA2, 23.8%of total variance explained). Numbers indicate the taxa examined (1: *G. minor*, 2: *G. chilospilus*, 3: *G. eurostus*, 4: *G. shaoi*, 5: *G. prionodon*, 6: *G. fimbriatus*, 7: *G. hepaticus*, 8: *G. flavimarginatus*, 9: *G. kidako*, 10: *G. pseudothyrsoideus*, 11: *G. thyrsoideus*, 12: *G. rueppelliae*, 13: *G. neglectus*, 14: *G. margaritophorus*, 15: *G. pictus*, 16: *E. polyzona*, 17: *E. nebulosa*, 18: *U. macrocephalus*, 19: *U. micropterus*, 20: *S. sathete*, 21: *P. cancrivorus,* and 22: *D. anguillare*). Numbers in blue belong to the Family Muraenidae and numbers in green represent the outgroup.

**Figure 3:**
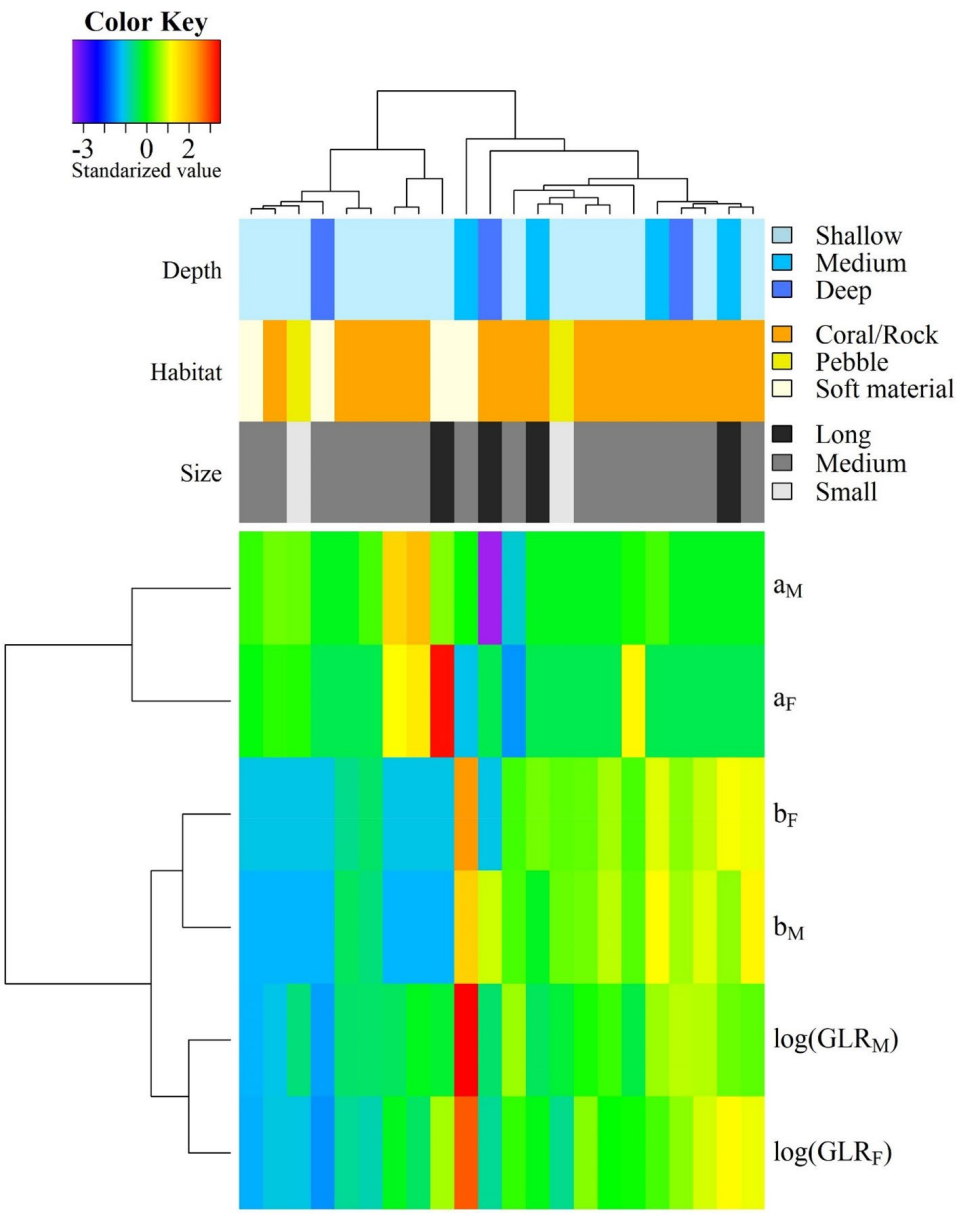
Heat-map of the standardized directional symmetry coefficients and habitat types (coral/rocky reefs, peddle beach, and seabed of soft materials), and maximum observed depth (shallow ≤ 50 m, medium: 50–100 m, and deep: > 100 m) and size categories (small: < 50 cm, medium (50–100 cm) and long (> 100 cm).

The directional asymmetry in the gonad length for 22 selected species was visualized by the biplot of principal component analysis (PCA), with the first two PCA axes explaining 85.6% of the total variation (Fig. 2). The first PCA axis is contributed mainly by the logged gonad length ratio and the slope of the relationship between the gonad length difference to the total length. In contrast, the second PCA axis is mostly the intercept (purple arrows in Fig. 3). Generally, the studied species were scattered randomly in the biplot, but two species stood out, *G. minor* (number 1) and yellow-edged moray *Gymnothorax flavimarginatus* (number 8). Two outgroup species (numbers in green) were also distributed close to the species in Muraenidae (numbers in blue).

Generally, the hierarchical cluster grouping of the directional asymmetry coefficients did not exhibit a strong and visible relationship to the habitat types, depth, and size classes, because these classes exhibited an intermingled pattern without a strong correspondence (Fig. 3). The taxonomic closeness, represented by the taxonomic distance based on the COI marker or the taxonomic distinctness index, did not play a significant role in this asymmetry as indicated by the Mantel test (9999 permutations, *p* = 0.625 and 0.535, respectively).

## Discussion

In this study, we examined nearly 3000 individuals from 22 eel species and found a directional asymmetry in the gonad lengths of the moray eel species (Muraenidae). The length of their gonads on the right side was significantly longer than that on the left side, leading to the rejection of our first hypothesis, no directional asymmetry. This was likely a unique and widely-occurring phenomenon in moray eels, because this phenomenon was observed in all 20 muraenids examined, but not in two outgroup species, *Pisodonophis cancrivorus* (Ophichthidae) and *Dysomma anguillare* (Synaphobranchidae). Moreover, rather than a consistent pattern, the species examined exhibited considerable variation in the degree of directional asymmetry in the gonad length (Tables 1, 2; Fig. 2), thus leading to the rejection of the second hypothesis, the same degree of directional asymmetry for all examined species. This variation in the degree of asymmetry was not affected by the major habitat types, depth, and size classes (Fig. 3). Therefore, the third hypothesis, no effects from the habitat types, depth, and size classes on the directional asymmetry, was retained.

Natural variation can lead to the asymmetry in the gonad morphology^15^. The asymmetry in the gonad morphology was considered as an abnormal phenomenon^14^ because it occurred in a minority (0 to 10% in 15) of the specimens. However, this study shows a different case that most of the moray eel specimens exhibit this directional asymmetry in the gonad length (Supplement material 5 for the scatter plots). Therefore, directional asymmetry in the gonad length might be the norm of the moray eel species examined and the possibility of natural variation can be ruled out.

Asymmetry in the gonad length (or weight) was occasionally observed in the teleost with one side being longer or heavier than the other one, and often related to somatic size and sex^11–13^. Asymmetry in gonad size was observed in 95% of the albacore (*Thunnus alalunga*) specimens examined of both sexes, with the right gonad larger than the left in 72% of the specimens and increased with increasing fork length^11^. Both sexes of the pollan (*Coregonus autumnalis*) showed directional asymmetry in gonad weight, with the left more prominent than the right in 70% of the specimens^12^. The frequency and degree of asymmetry are size-independent in males but became progressively more marked in females of > 120 g in weight. The gonad at the left side of the ayu, *Plecoglossus altivelis* was consistently longer and heavier than on the right side^13^. This asymmetry also became more pronounced with increasing age, especially in females.

Observed directional asymmetry in the gonad length may lead to some functional differences^31^. For example, the shorter side might contribute fewer gametes^32^. As the mature stages advanced, fully matured females might encounter a balance issue in the left–right direction in the spawning season because the egg sizes become larger and heavier, especially in *Gymnothorax minor* which showed the most extreme asymmetry. However, the directional asymmetry had, if there is any, only limited adaptive disadvantage in survival because these moray eels have persisted. Since the gonads are located in the abdominal cavity, the asymmetry would lead to little external morphological difference and therefore, the selection resulting from the morphological difference can hardly work. Fully matured gonads were found on both sides for most of the moray species examined^33,34^ and therefore, the smaller contribution from the shorter side can be compensated by the longer side^35^.

We hypothesize that observed directional asymmetry in moray eels is a by-product of evolutionary history^8,36^, rather than an adaptation for better survival^37^, or a consequence of environmental stresses^6^. First, all morays exhibited this asymmetry, while two outgroup species from different families did not, suggesting this asymmetry likely to be a by-product of the evolution of Muraenidae, which involves many unique morphological adaptations, such as highly modified gill arches that the fourth arc can be projected anteriorly^38^. Second, as argued earlier, the directional asymmetry might lead to little adaptive disadvantage in survival. Third, as represented by the mosaic patterns in Fig. 3, the degree of asymmetry was not related to major habitat types and depth classes. This suggested that environmental factors had played insignificant roles in shaping observed directional asymmetry.

This study provides new puzzle pieces for the full picture of the directional asymmetry in gonad size observed in other vertebrates, particularly in birds^39–41^. Witschi^41^ hypothesized that the asymmetry in the oviducts of female birds might have evolved as an adaptation to the aviatic life for reduced body weight and enhanced air dynamics. This is probably not the major factor in the marine environment where the density of water allows organisms to attain neutral buoyancy^42^. The packaging hypothesis states that asymmetry in gonads might reflect space constraints within the body cavity^35^, which is also not sufficient to explain the observed asymmetry in the moray eels because their elongated body provides enough room in the abdomen for the gonads for both sides (Fig. 1). Sexual selection^37,43^ or a by-product of the development of the secondary jaw^5,38^ are two possible explanations for observed asymmetry in the moray eels, which can be tested in future studies.

## Materials and methods

### Collection of specimens

The moray eels used in this study were collected from eastern and northeastern Taiwan from 2003 to 2008 by various methods. Using bottom long-lines operating over eastern Taiwanese rocky coasts, most specimens were collected by collaborating fishermen. Some specimens were collected from the by-catch of trawlers operating over the northeastern Taiwan coast. Specimens were also collected from nearshore tidal pools by hook-and-line or anesthesia (clove oil). The specimens were transported back to the laboratory and frozen at − 20 °C.

After defrosting, total lengths (to 0.1 cm) and weights (to 0.1 g) of the specimens were measured. Then both sides of the gonads were removed after dissection. The left and right sides are defined when the fish head up and belly down (Fig. 1). The length of the gonad was measured to the nearest 0.1 mm and the weight was measured to the nearest 0.01 g. The sexes were determined by macroscopical examination of the gonads and later double-checked by microscopical examination^33,34^. We observed that both gonad length and weight exhibit left–right asymmetry. We use the gonad length, not the gonad weight, to represent the left–right asymmetry for being more robust to variation in sampling months.

Information about the major habitats, observed maximum length, and maximum depth of the studied taxon was obtained from FishBase^25^. The observed maximum length value from FishBase was replaced by the maximum observed length in our database if the latter was larger than that in the FishBase. The major habitats were categorized into three types: coral/rocky reef, pebble beach, and seabed of soft materials. The maximum observed lengths were categorized into three size classes: small (< 50 cm), medium (50–100 cm), and long (> 100 cm), and the maximum observed depths into three categories: shallow (≤ 50 m), medium (50–100 m), and deep (> 100 m).

### Representing the directional asymmetry, quantifying the degree of directional asymmetry, and testing the effects of total length and sex

Two indicators represent the directional asymmetry in the gonad length of the species examined. The first is the ratio of the gonad length of two sides (right divided by left) and the second is the gonad length difference (right minus left).

Ratios are commonly used as a measure without strong effects on the total length^12,44^. Ratios deviating from 1 indicate the existence of directional asymmetry in the gonad length; the larger the deviation the stronger the degree of directional asymmetry. The gonad length ratio was log-transformed and the effect of sex on the gonad length ratio was tested by a linear regression model with the Gaussian likelihood function. Two models were constructed: null model: the ratio was not affected by the sex, and an alternative model where the ratio was affected by the sex. Akaike information criterion (AIC) values for both models were calculated and used for model comparison. However, according to Burnham and Anderson^45^, a difference in AIC value (δAIC, the AIC value between the AIC value from a candidate model and the lowest AIC value) < 2.0 between the two models indicates an insignificant difference. Therefore, we selected the best model according to the following criteria: (1) the lowest AIC value with a δAIC value > 2.0, (2) if δAIC value < 2.0, then we select the one with the fewest parameters following the parsimony principle. The significance level (α) was set at ≤ 0.05. All the computation was completed in *R* (version 4.1.3)^46^

It is also common to use the difference in a given trait between the two sides to represent the directional asymmetry^2,3^. Moreover, the gonad length ratio is still not completely independent of the total length (Supplement material 4), while the gonad length difference can explicitly model the linear effect of total length on gonad length^2^. Therefore, multiple linear regression was applied to test the effects of total length and sex. Five models were constructed, as visualized in Supplement material 6: Null model: the gonad length difference was a constant (intercept-only model). In this model, a directional asymmetry exists if the intercept > 0. Model 1: the gonad length difference increased with the total length without differences between sexes. Model 2: the gonad length difference increased with total length, and the difference in intercepts represented the difference between sexes. Model 3: the gonad length difference increased with total length, and the difference in slopes represented the difference between sexes. Model 4: the gonad length difference increased with total length, and the difference in intercepts and slopes represented the difference between sexes. The best model was selected by the criteria previously described.

### Taxonomic distances between species

Two approaches were applied to represent the taxonomic closeness among selected species. The first approach was to calculate a taxonomic distance based on the mitochondrial cytochrome oxidase subunit I (COI) sequence as done in Lin et al.^47^. The COI sequences were obtained from GenBank (www.ncbi.nlm.nih.gov/genbank/) where sequences for 20 species were available. One to six sequences were downloaded for each species and the sequences of specimens from Taiwan were preferred (see Supplement material 7 for detailed records of downloaded COI sequences). Multiple sequence alignment was applied to the raw COI sequences from GenBank using the ClustalW algorithm in the *R* package *msa*^48^. The Kimura two-parameter (K2P) distances between the specimens were calculated using the *R* package *ape*^49^. Then the average distances between the 20 species were calculated using *R* package *vegan*^50^ and used as one measure of the taxonomic closeness.

The second approach is to use the taxonomic distinctness index, the average path length through a standard Linnaean tree^51^ as a measure of taxonomic closeness. A Linnaean taxonomic tree (Family, Genus, and Species) of these 20 species whose COI sequences are available in Genbank was constructed and the averaged taxonomic distinctness index among species was calculated using the *R* package *vegan*.

### Multivariate analysis

Correlation-based principal component analysis was applied to visualize the coefficients representing the directional symmetry in gonad length (i.e., the gonad length ratios, and the intercepts and slopes of the linear relationship between the gonad length difference and total length). These coefficients were standardized with a mean of zero and a standard deviation of 1. We applied cluster analysis based on the Euclidean distance of standardized coefficients representing the directional symmetry in gonad length with Ward’s algorithm^52^. The heatmap was produced to simultaneously reveal the hierarchical cluster structures over the coefficients and the species with different categories (i.e., habitat types, depth, and size classes). Mantel test with 9999 permutations^53^ was applied to test the non-parametric correlation (Kendall’s τ) between the distance matrix of directional asymmetry coefficients and distance matrices representing the taxonomic closeness using *R* package *vegan*.

## Supplementary Information

Supplementary Information.

## Data Availability

The datasets analyzed during the current study are available from the corresponding author on reasonable request.
