# Supplementary material for: Directional asymmetry in gonad length indicates moray eels (Teleostei, Anguilliformes, Muraenidae) are “right-gonadal”
Source: Sci Rep. 2023 Feb 20;13:2963. doi: 10.1038/s41598-023-29218-3 (PMC9941099; doi:10.1038/s41598-023-29218-3)
Supplement: Supplementary file 1 — Supplementary Information. [file 41598_2023_29218_MOESM1_ESM.docx]

**Supplement material 1**. Sample sizes (N), mean total length (L_T_, cm, ± SD), total somatic mass (M_T_, g), and the ratio of the right gonad length divided by the length of the left gonad (GLR), by species and sex (F = female and M = male).

| **Species** | **N** | | L_T_ | | M_T_ | |
| --- | --- | --- | --- | --- | --- | --- |
|  | **F** | **M** | **F** | **M** | **F** | **M** |
| **Muraenidae species** |  |  |  |  |  |  |
| *Gymnothorax minor* | 303 | 349 | 41.3 ± 6.2 | 45.3 ± 5.6 | 76 ± 44 | 105 ± 46 |
| *Gymnothorax chilospilus* | 39 | 446 | 26.8 ± 3.0 | 31.1 ± 3.7 | 24 ± 11 | 39 ± 16 |
| *Gymnothorax eurostus* | 60 | 254 | 35.0 ± 3.8 | 41.4 ± 5.8 | 74 ± 27 | 138 ± 66 |
| *Gymnothorax shaoi* | 70 | 98 | 45.3 ± 6.2 | 47.1 ± 8.1 | 140 ±67 | 163 ± 101 |
| *Gymnothorax prionodon* | 76 | 70 | 49.3 ± 11.3 | 50.7 ± 11.5 | 174 ±154 | 203 ± 174 |
| *Gymnothorax fimbriatus* | 68 | 59 | 53.9 ± 12.6 | 54.4 ± 15.1 | 255 ± 188 | 313 ± 296 |
| *Gymnothorax hepaticus* | 48 | 52 | 54.4 ± 10.5 | 56.2 ± 10.9 | 220 ± 138 | 258 ± 157 |
| *Gymnothorax flavimarginatus* | 33 | 46 | 53.4 ± 17.6 | 47.5 ± 12.9 | 367 ± 503 | 204 ± 185 |
| *Gymnothorax kidako* | 28 | 53 | 42.6 ± 14.3 | 42.0 ± 7.7 | 191 ± 264 | 95 ± 62 |
| *Gymnothorax pseudothyrsoideus* | 38 | 37 | 48.6 ± 12.7 | 42.8 ± 8.7 | 259 ± 237 | 136 ± 101 |
| *Gymnothorax thyrsoideus* | 24 | 41 | 53.0 ± 7.6 | 58.4 ± 7.1 | 233 ± 188 | 322 ± 170 |
| *Gymnothorax rueppelliae* | 21 | 30 | 49.6 ± 8.4 | 51.2 ± 10.3 | 176 ± 95 | 212 ± 177 |
| *Gymnothorax neglectus* | 30 | 27 | 53.5 ± 12.1 | 50.9 ± 12.4 | 199 ± 155 | 191 ± 160 |
| *Gymnothorax margaritophorus* | 11 | 32 | 34.4 ± 6.9 | 44.1± 9.3 | 46 ± 26 | 127 ± 87 |
| *Gymnothorax pictus* | 16 | 12 | 50.7 ± 12.2 | 55.8 ± 17.5 | 235 ± 159 | 322 ± 352 |
| *Echidna polyzona* | 63 | 72 | 43.5 ± 7.5 | 45.6 ± 5.6 | 146 ± 83 | 177 ±95 |
| *Echidna nebulosi* | 19 | 36 | 42.8 ± 10.3 | 48.4 ± 8.6 | 113 ± 69 | 168 ± 102 |
| *Uropterygius macrocephalus* | 20 | 62 | 28.5 ± 5.0 | 33.3 ± 4.1 | 28 ± 14 | 48 ± 23 |
| *Uropterygius micropterus* | 32 | 24 | 23.2 ± 4.5 | 24.6 ± 4.7 | 9 ± 6 | 12 ± 7 |
| *Strophidon sathete* | 61 | 21 | 103.6 ± 17.8 | 128.6 ± 25.9 | 417 ± 295 | 679 ± 286 |
| **Outgroup species** |  |  |  |  |  |  |
| *Pisodonophis cancrivorus* | 33 | 13 | 54.4 ± 12.0 | 39.8 ± 4.5 | 117 ± 93 | 31± 13 |
| *Dysomma anguillare* | 24 | 8 | 55.6 ± 10.1 | 50.3 ± 6.2 | 215 ± 122 | 115 ± 36 |

**Supplement material 2**. AIC difference (δ, AIC values – minimum AIC value) for the null model without sexual difference in GLR (δ_0_) and for the alternative model (with sexual difference in GLR, δ_1_) and estimated parameters (in natural logarithm, p-values in parenthesis) of the selected model (in bold), based on the following the criteria: (1) low AIC difference values < 2 and (2) with the fewest number of parameters. Supplement material

| **Species** | δ_0_ | δ_1_ |
| --- | --- | --- |
| *G. minor* | 354.4 | **0** |
| *G. chilospilus* | **0** | 0.5 |
| *G. eurostus* | 15 | **0** |
| *G. shaoi* | 83.6 | **0** |
| *G. prionodon* | 20 | **0** |
| *G. fimbriatus* | 8.2 | **0** |
| *G. flavimarginatus* | 41.6 | **0** |
| *G. hepaticus* | 10.4 | **0** |
| *G. kidako* | 12.5 | **0** |
| *G. pseudothyrsoideus* | 16 | **0** |
| *G. thyrsoideus* | **0** | 1.1 |
| *G. rueppelliae* | 9 | **0** |
| *G. neglectus* | **0** | 0.7 |
| *G. margaritophorus* | **0.2** | 0 |
| *G. pictus* | **0** | 0.8 |
| *E. polyzona* | **1.6** | 0 |
| *E. nebulosa* | **0** | 1.7 |
| *U. macrocephalus* | 6.4 | **0** |
| *U. micropterus* | 10.7 | **0** |
| *S. sathete* | 6.7 | **0** |
| *P. cancrivorus* | **0** | 1.2 |
| *D. anguillare* | **0** | 0.9 |

Abbreviation for genera: *G*. = *Gymnothorax*, *E*. = *Echidna*, *U.* = *Uropterygius*, *P*. = *Pisodonophis*, *S*. = *Strophidon* and *D*. = *Dysomma*.

**Supplement material 3** Diagrams showing models representing different hypotheses about the relationship between the gonad length difference (right- left), as a measure of directional asymmetry, to the total length and the sexes. Model 0 intercept only: directional asymmetry exists but independent of the length. It has a special case when the intercept equals zero (Model 0.0), which indicates no directional asymmetry. Model 1: directional asymmetry with the length effect. Model 2: directional asymmetry with the length effect and different intercepts by sexes. Model 3: directional asymmetry with the length effect and different slopes by sexes. Model 4: directional asymmetry with the length effect and different intercepts and slopes by sexes.


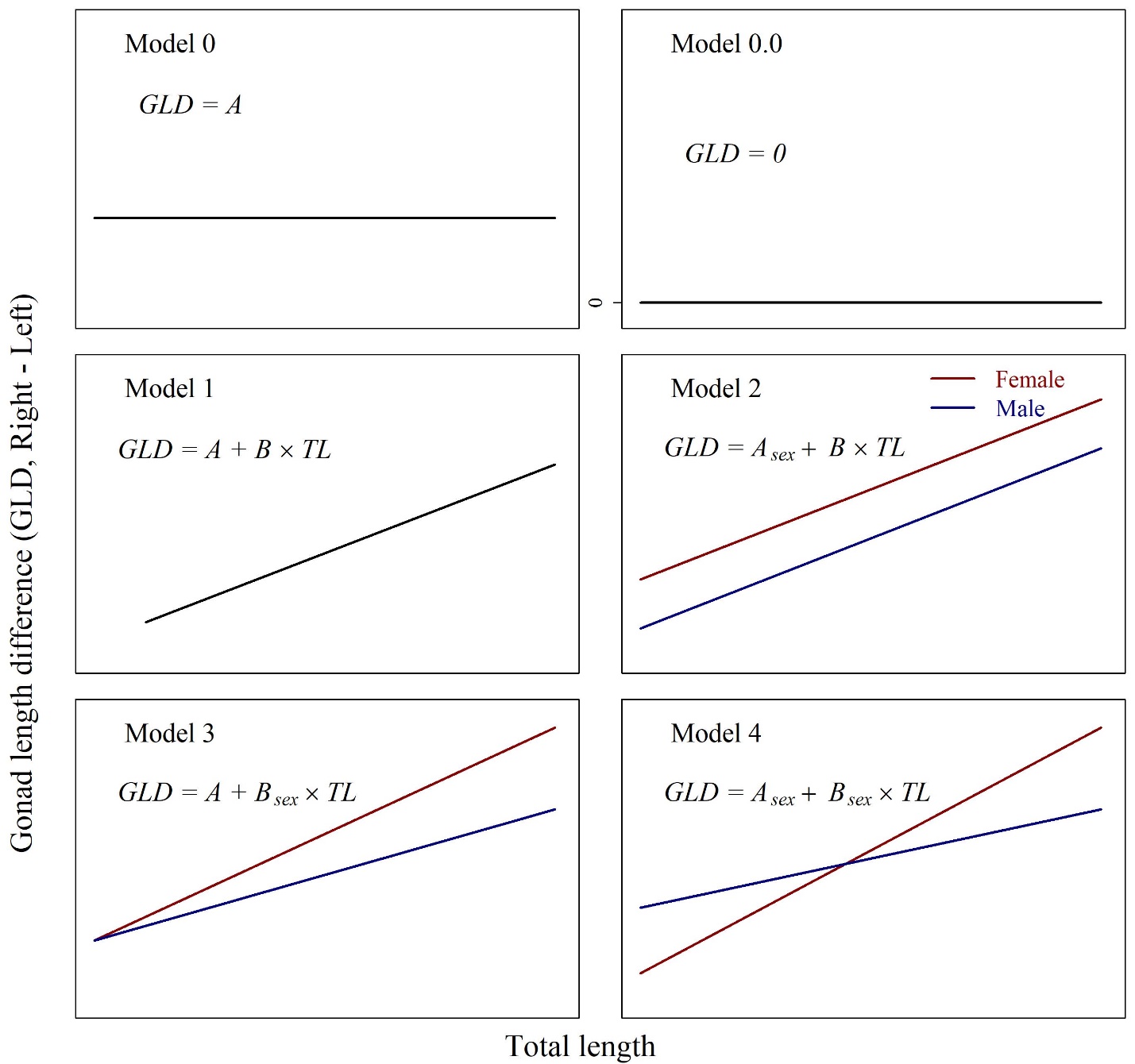


**Supplement material 4**. AIC difference (AIC values – minimum AIC value) for different relationships between gonad length difference (GLD, right – left) and total length and sex. M0 is the intercept only model, M2 is the total-length only model, M3 to M5 test the sex effect (detailed formula of model M1 to M4 refer to Fig. 2). AIC difference values < 2 are marked in bold for having insignificant differences one another (Buchman, 2002). The best model is in bold. Supplement

| Species | **M0** | **M1** | **M2** | **M3** | **M4** |
| --- | --- | --- | --- | --- | --- |
| *G. minor* | 371.5 | 54.8 | 8.2 | 2.7 | **0.0** |
| *G. chilospilus* | 18.3 | 18.0 | **0.0** | 1.3 | 1.0 |
| *G. eurostus* | 16.3 | 5.8 | **0.3** | 0.0 | 2.0 |
| *G. shaoi* | 64.5 | 6.8 | **0.2** | 1.6 | 0.0 |
| *G. prionodon* | 103.6 | **0.0** | 1.7 | 1.7 | 3.7 |
| *G. fimbriatus* | 12.0 | 6.6 | 3.9 | **1.3** | 0.0 |
| *G. flavimarginatus* | 54.7 | 4.4 | **0.0** | 0.8 | 2.0 |
| *G. hepaticus* | 31.1 | 11.3 | 6.3 | 3.4 | **0.0** |
| *G. kidako* | 25.5 | **0.0** | 1.9 | 2.0 | 1.3 |
| *G. pseudothyrsoideus* | **0.5** | 2.4 | 1.4 | 0.0 | 0.2 |
| *G. thyrsoideus* | **0.0** | 0.1 | 1.6 | 1.6 | 3.6 |
| *G. rueppelliae* | 21.8 | **0.0** | 2.0 | 2.0 | 4.0 |
| *G. neglectus* | 41.6 | 14.7 | 3.0 | **0.0** | 0.8 |
| *G. margaritophorus* | 13.1 | **1.4** | 3.4 | 3.3 | 0.0 |
| *G. pictus* | 28.2 | 21.7 | 4.9 | **0.0** | 1.1 |
| *E. polyzona* | **0.6** | 2.4 | 0.0 | 0.5 | 1.7 |
| *E. nebulosa* | 6.9 | **0.0** | 1.8 | 1.9 | 3.8 |
| *U. macrocephalus* | **0.0** | 1.4 | 1.6 | 1.4 | 3.4 |
| *U. micropterus* | 14.0 | **0.0** | 0.5 | 0.3 | 2.2 |
| *S. sathete* | 37.0 | 36.0 | **0.0** | 4.4 | 1.0 |
| *P. cancrivorus* | **0.0** | 2.0 | 4.0 | 4.0 | 5.7 |
| *D. anguillare* | **0.0** | 0.7 | 1.7 | 1.3 | 1.8 |

**Supplement material** **5**. Scatter plots of the gonad length on total length, by body side (green = right and purple = left) and sex (female and male) for selected moray eel and outgroup taxa.


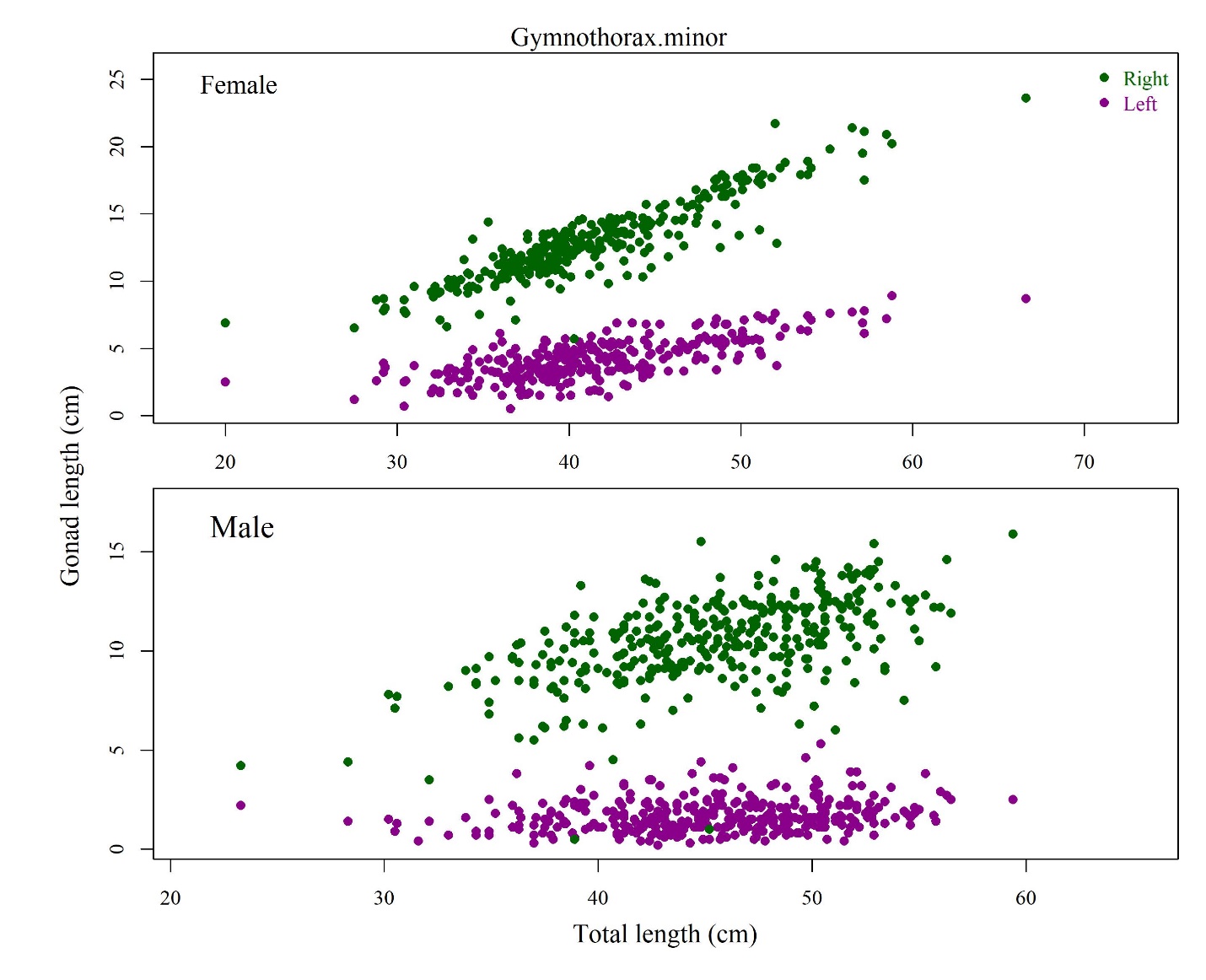


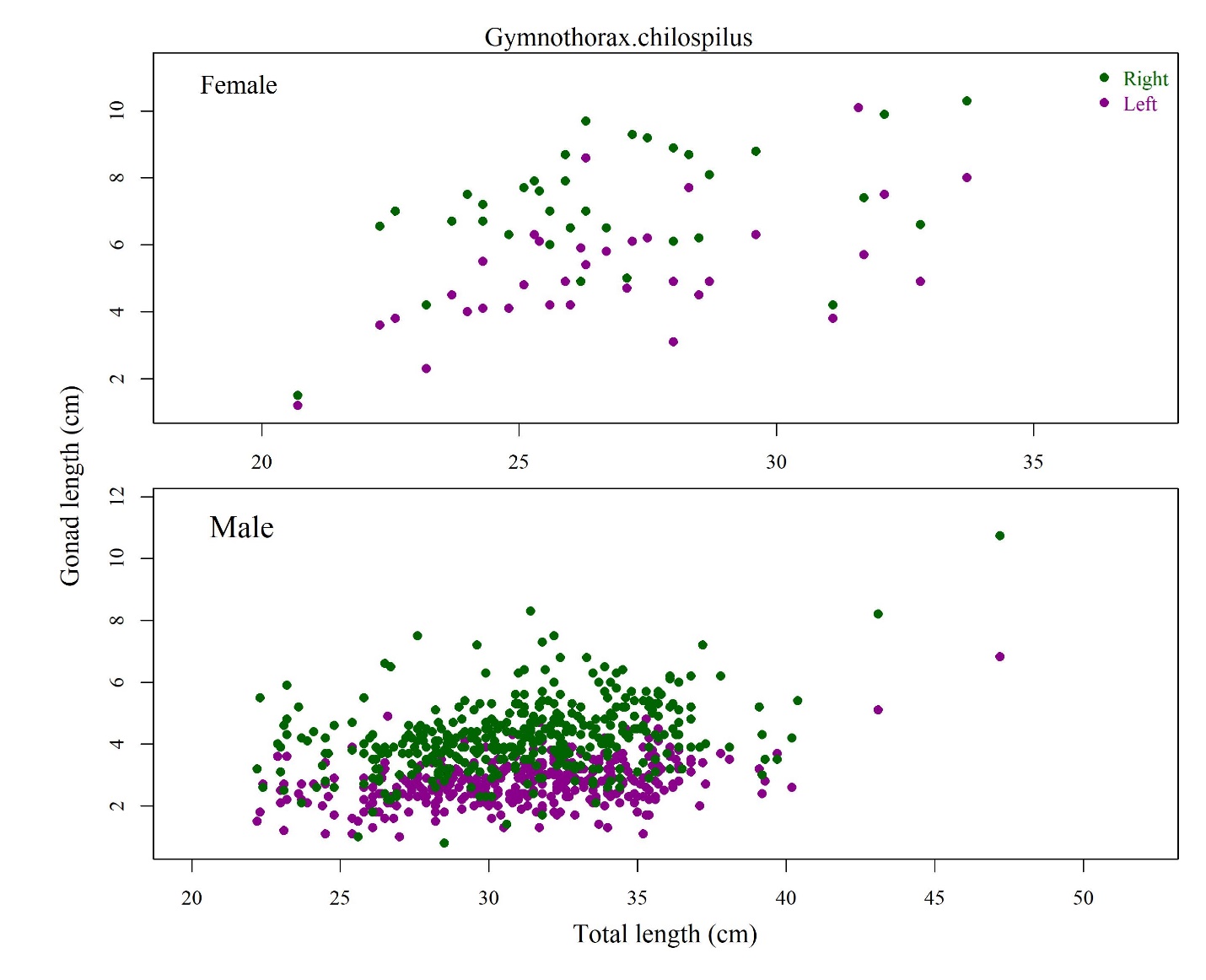


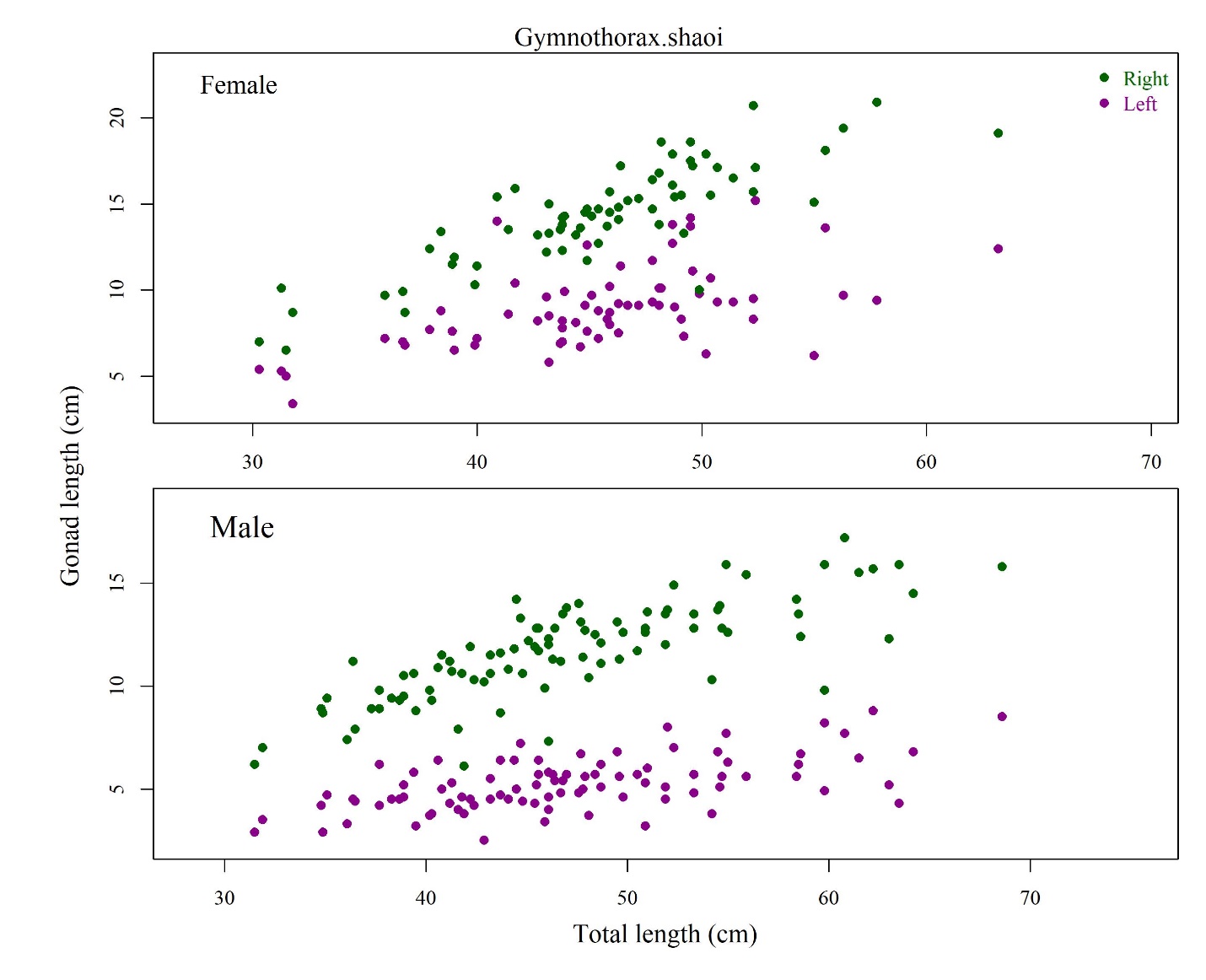


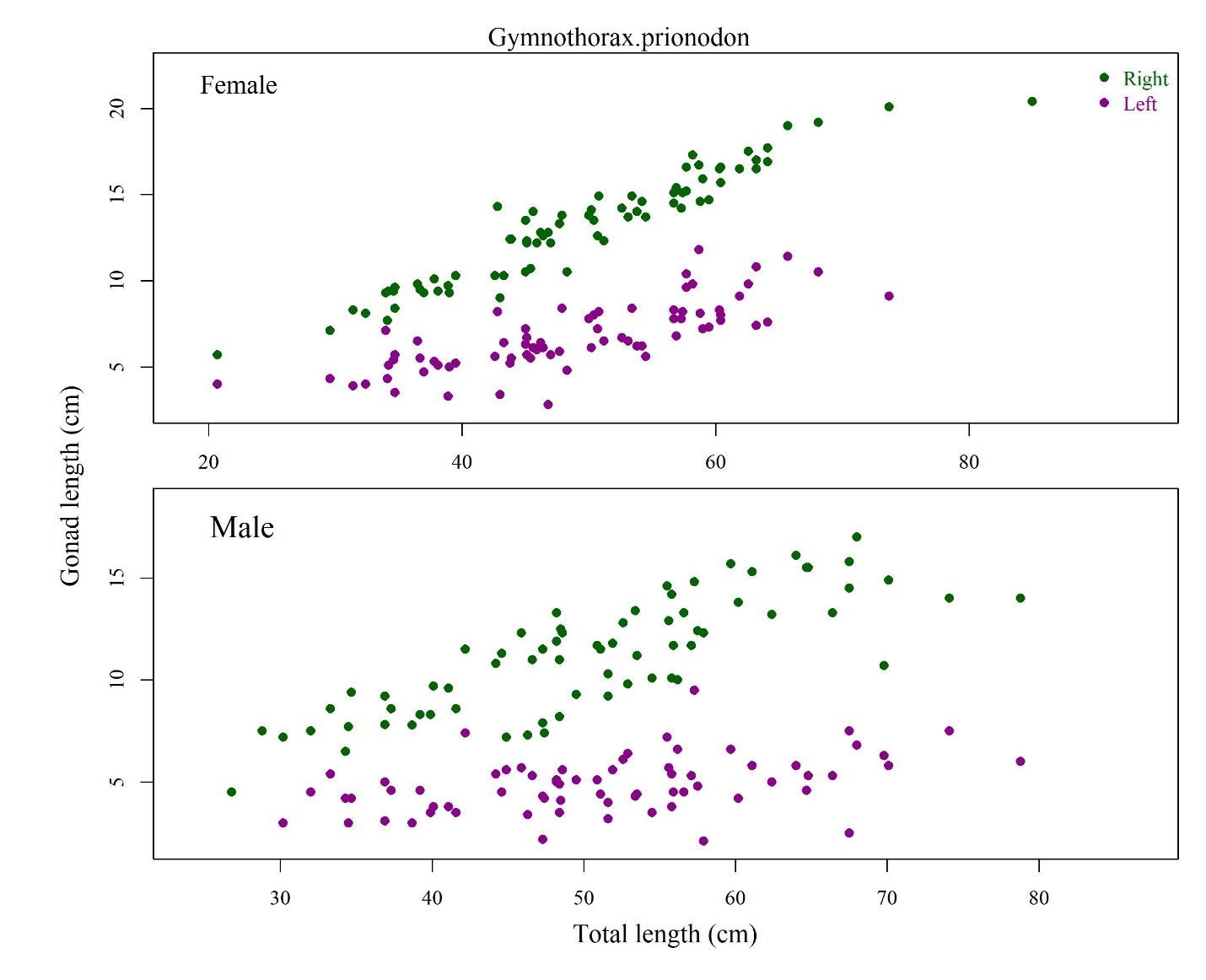

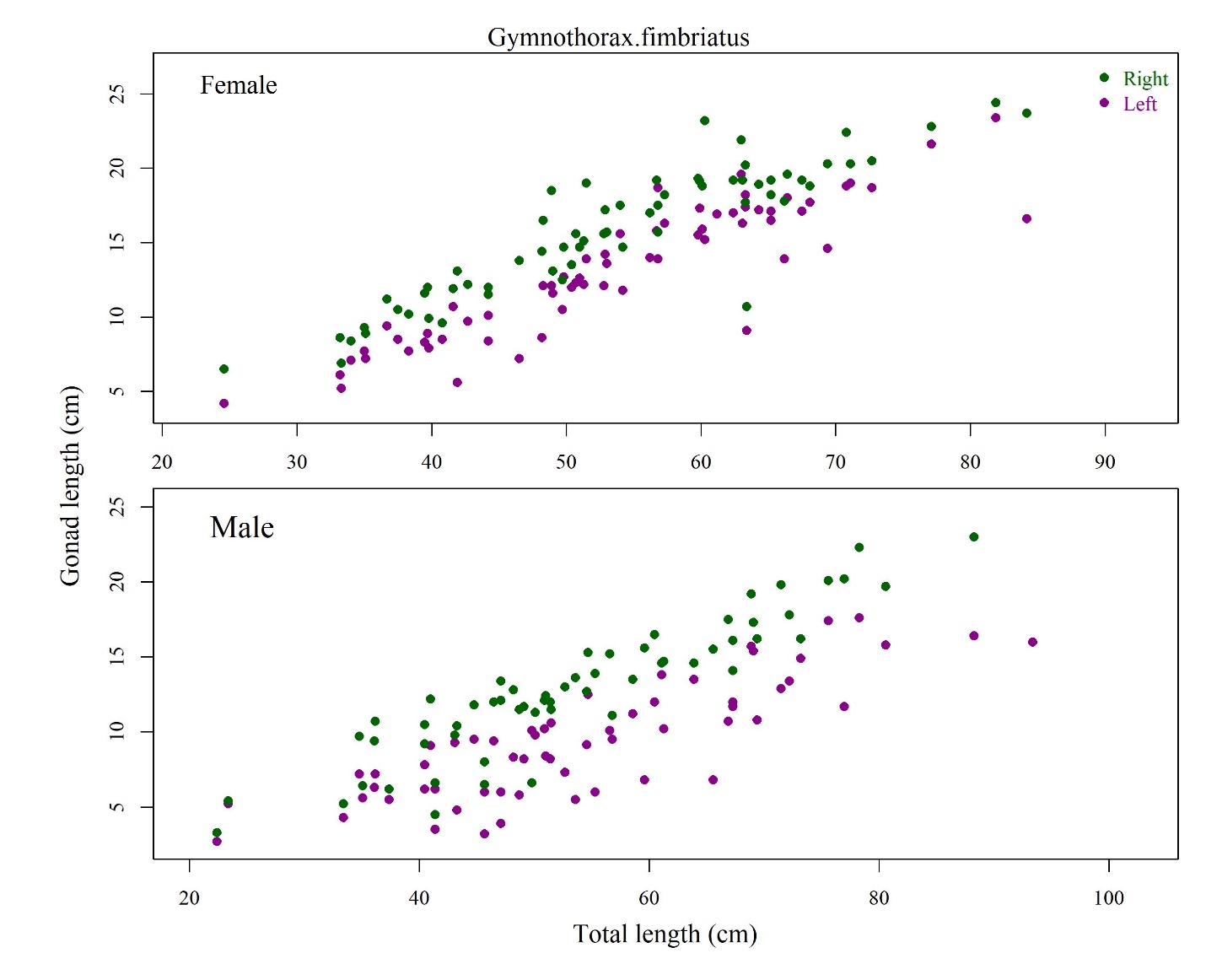


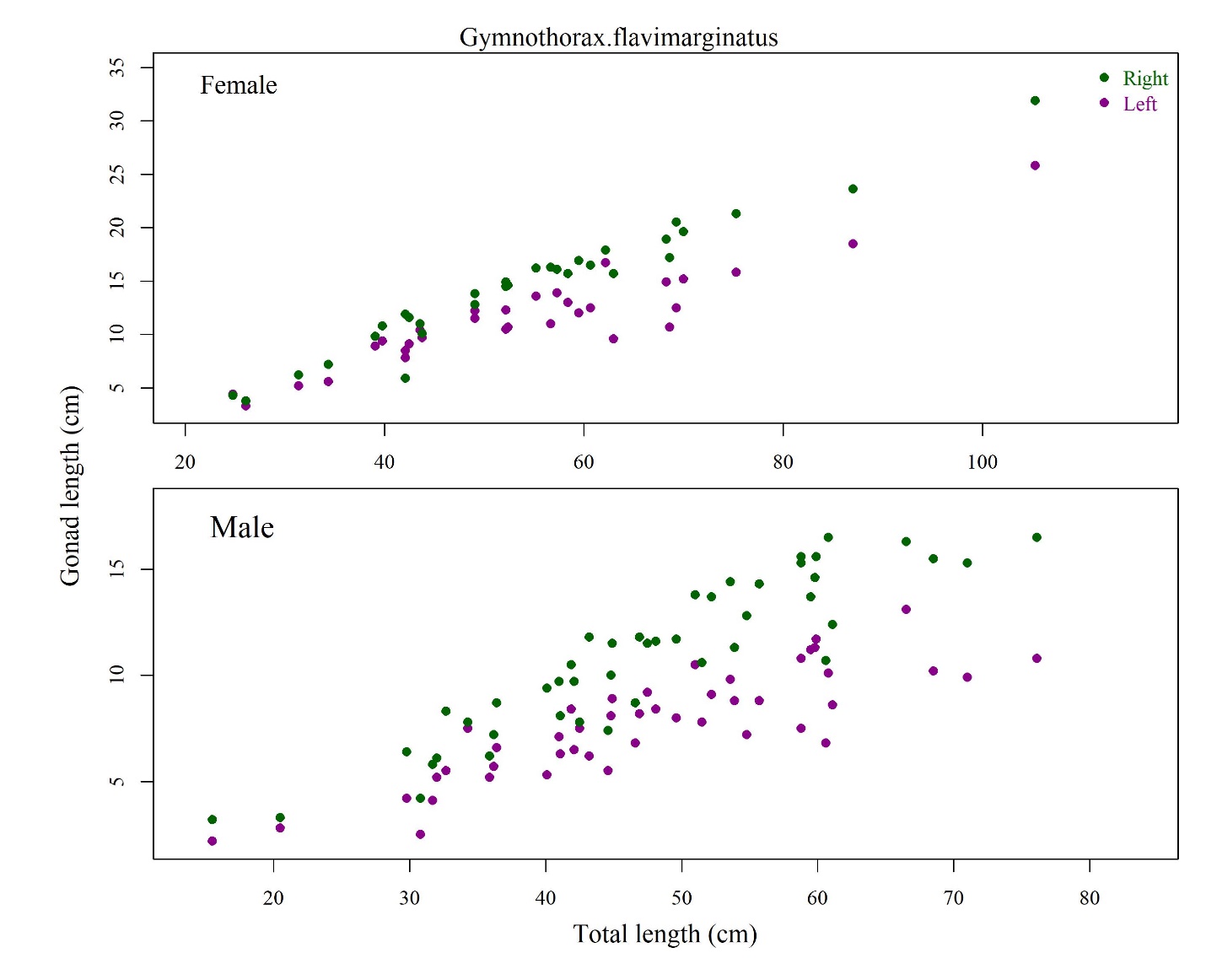


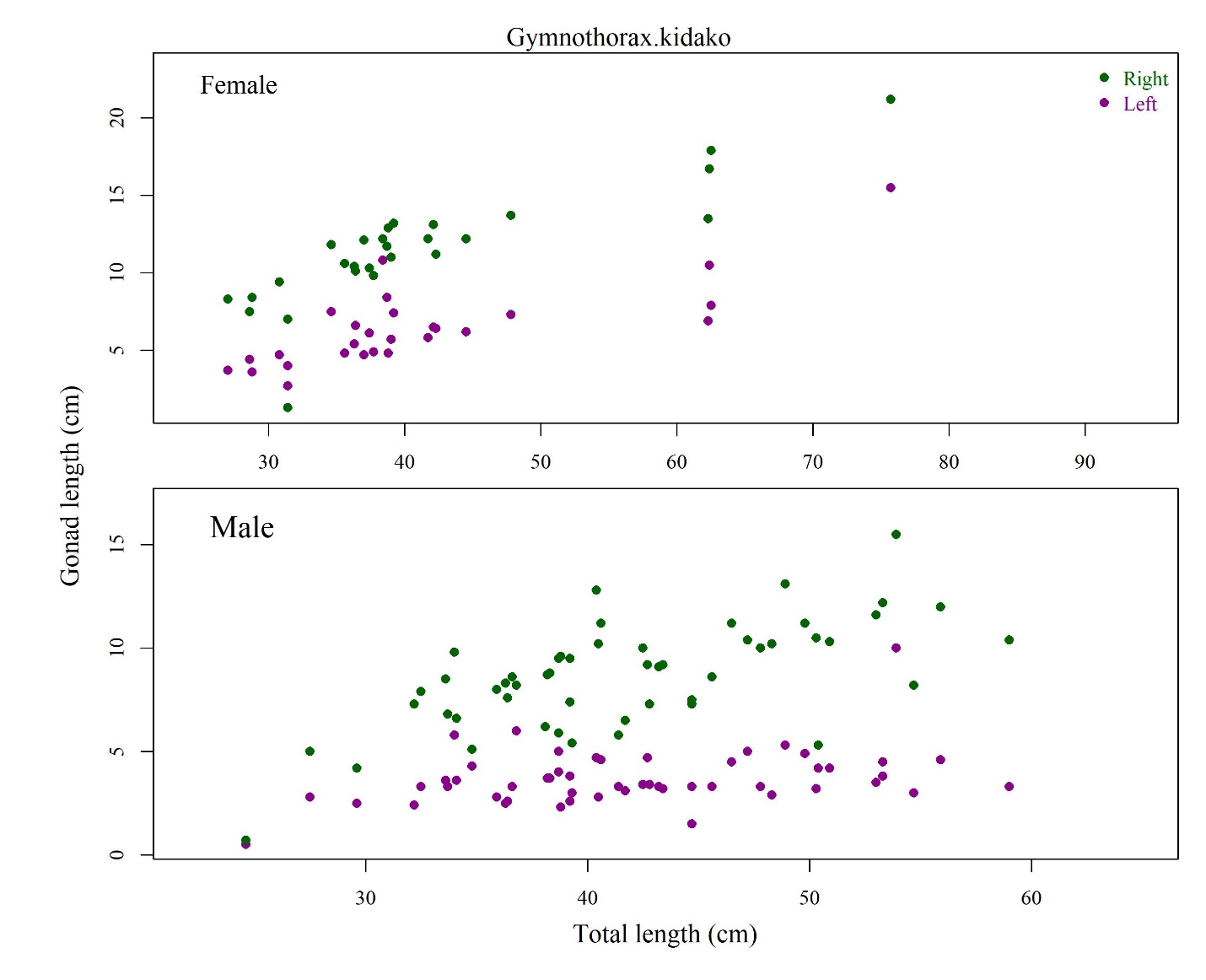


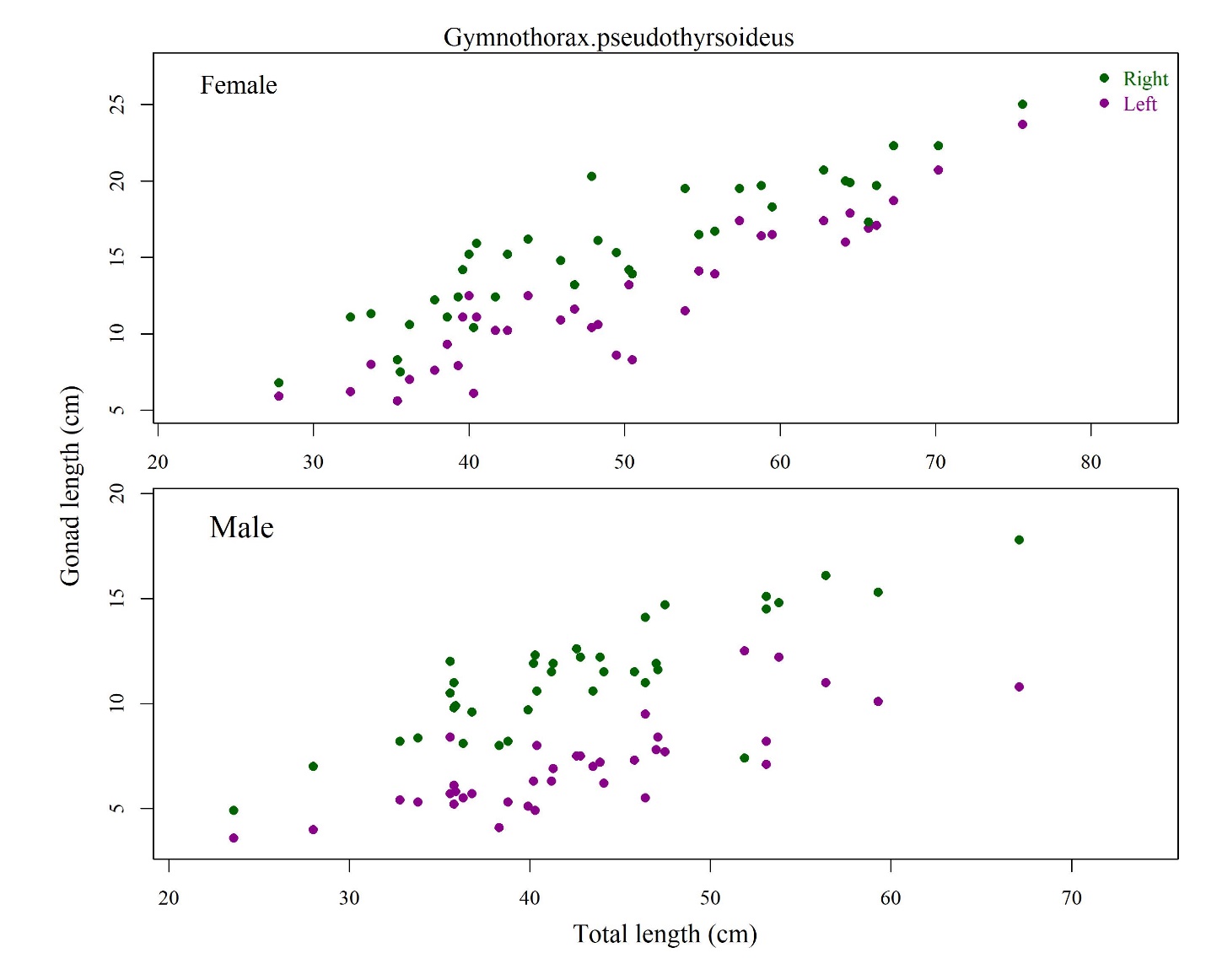


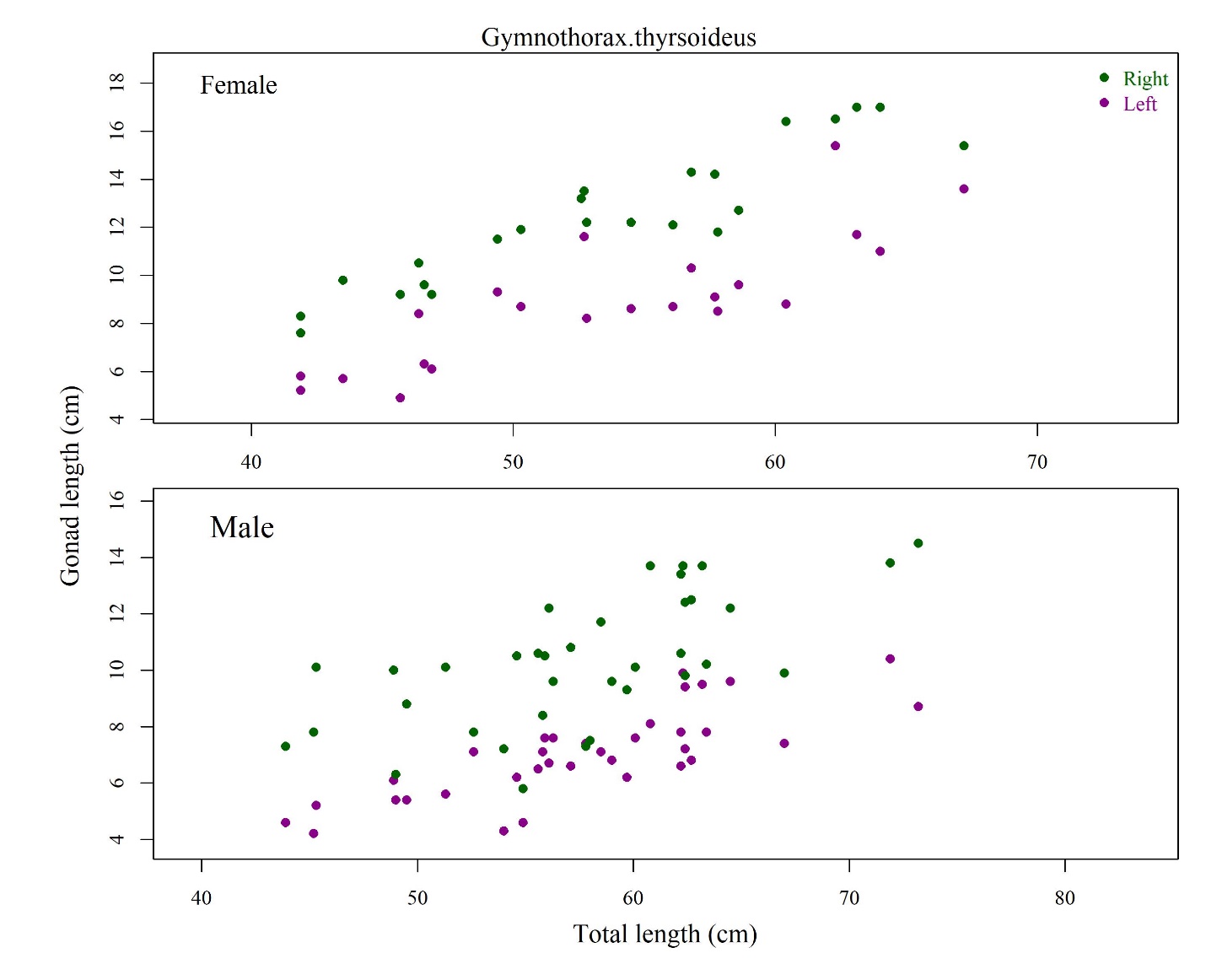


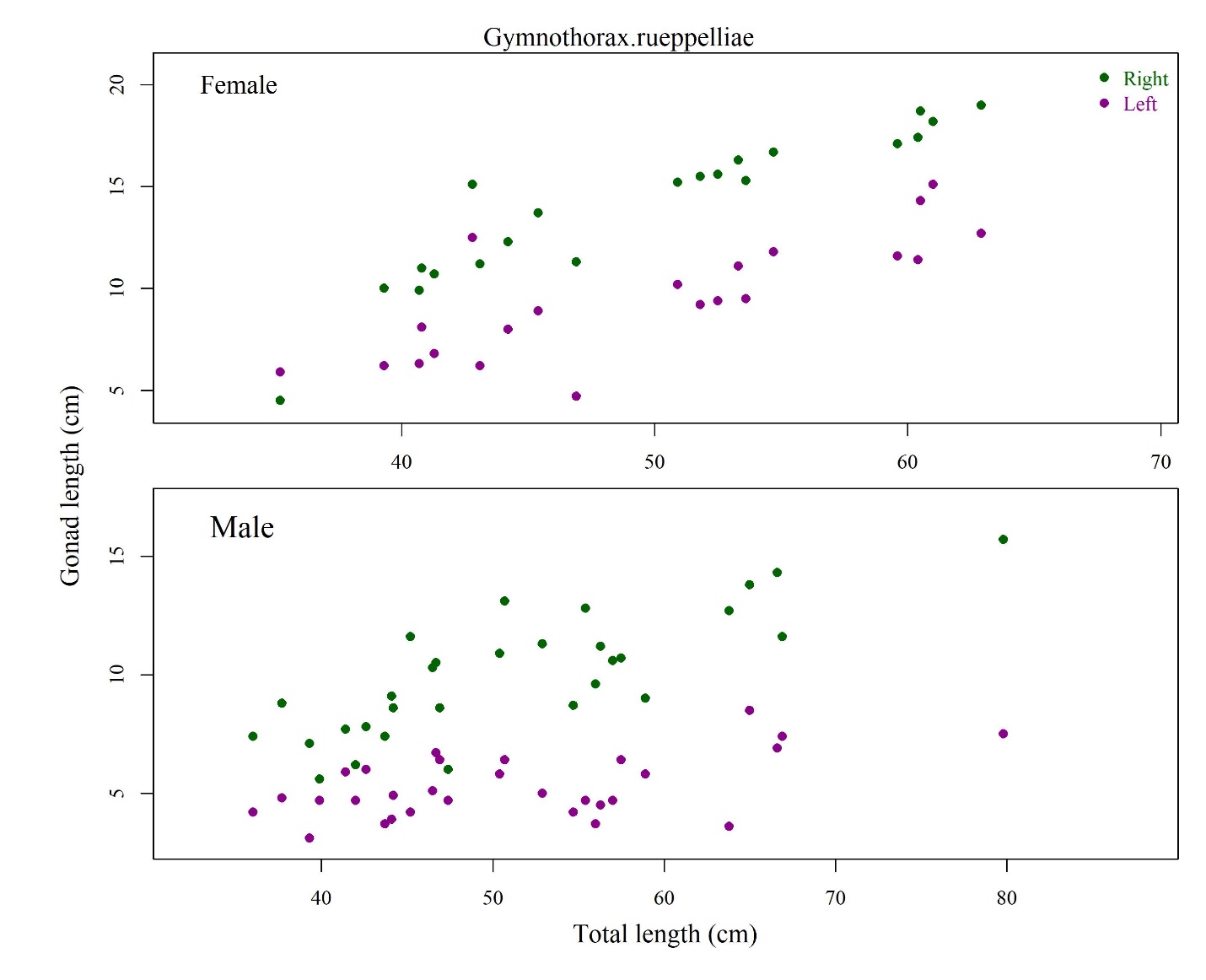


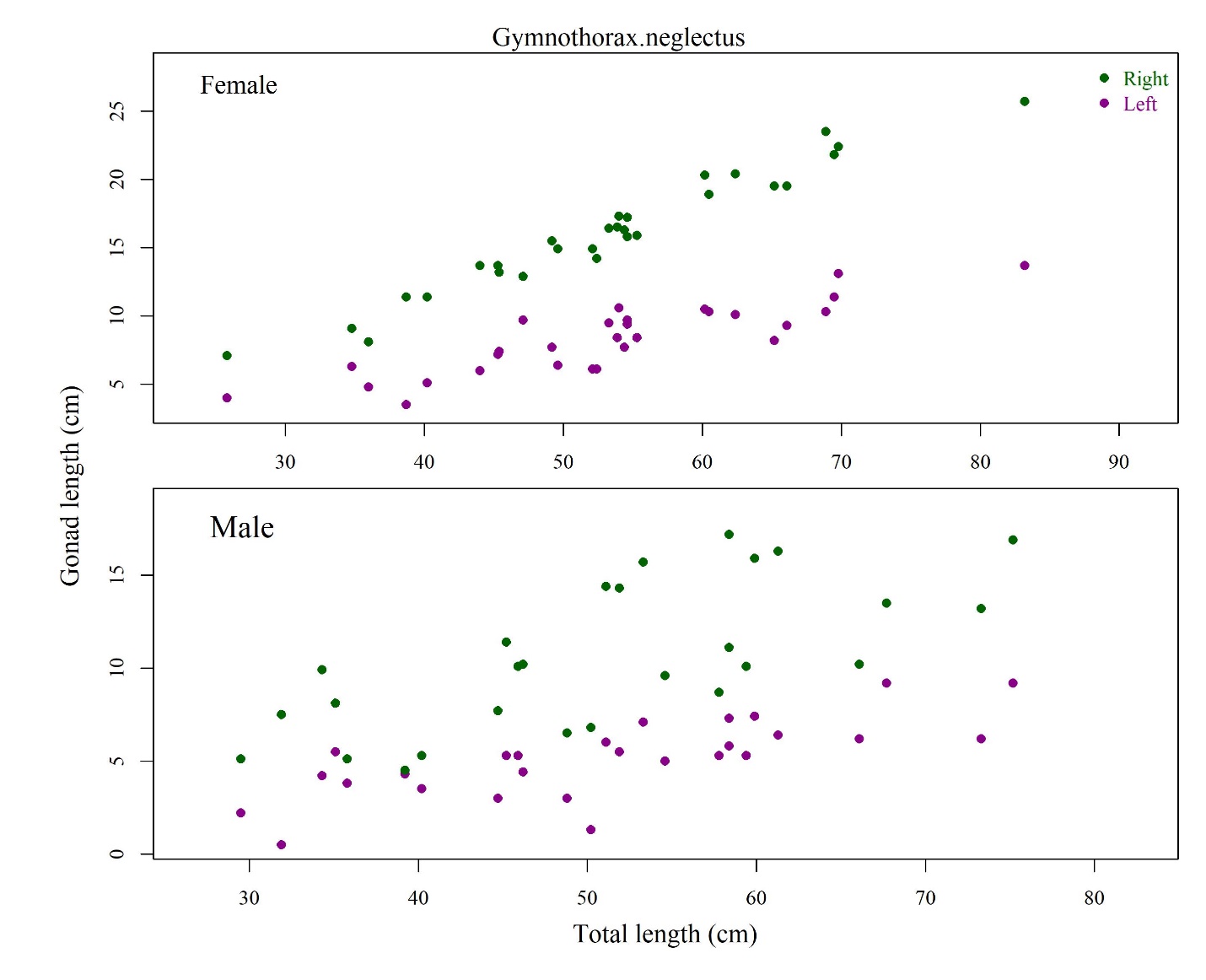


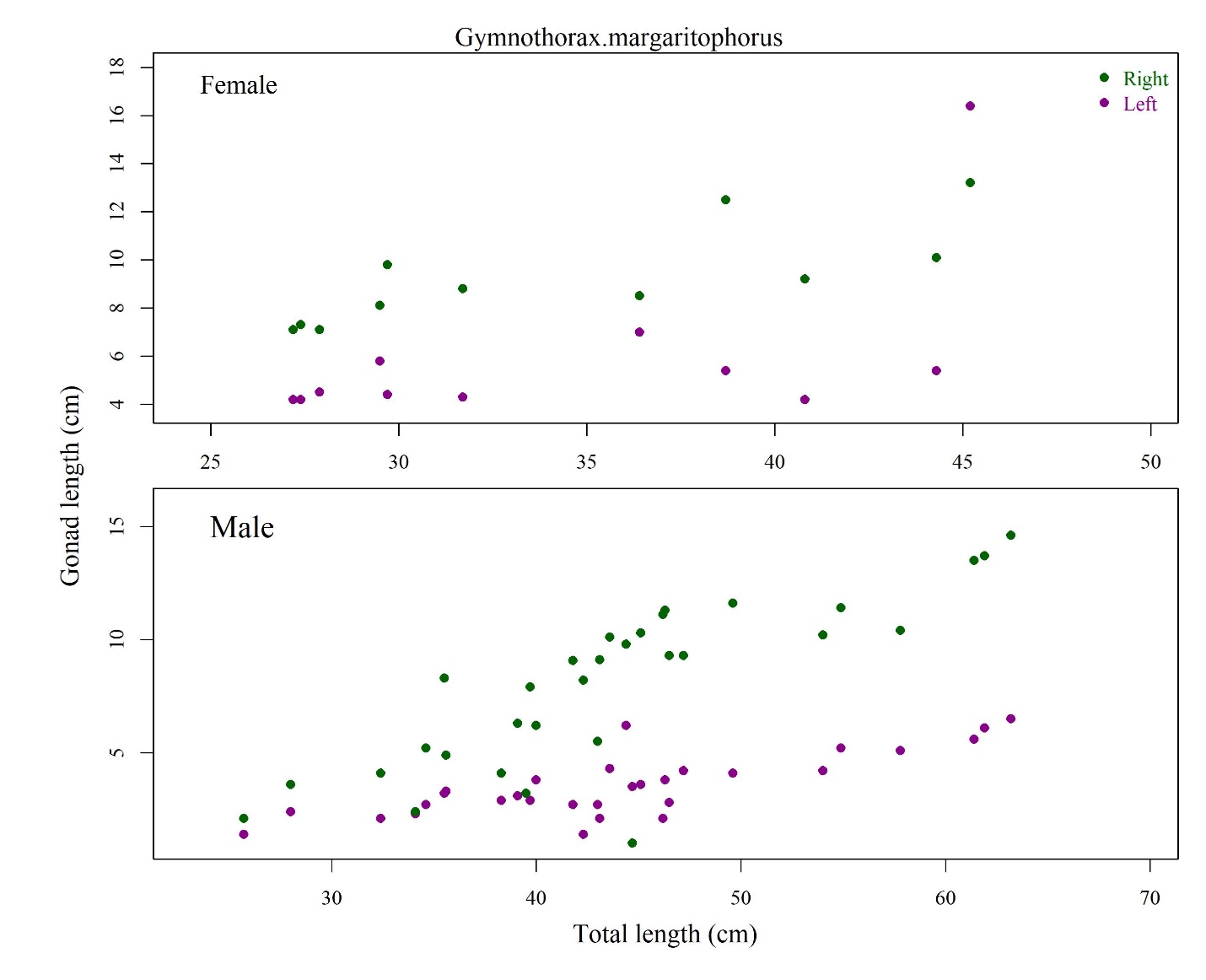


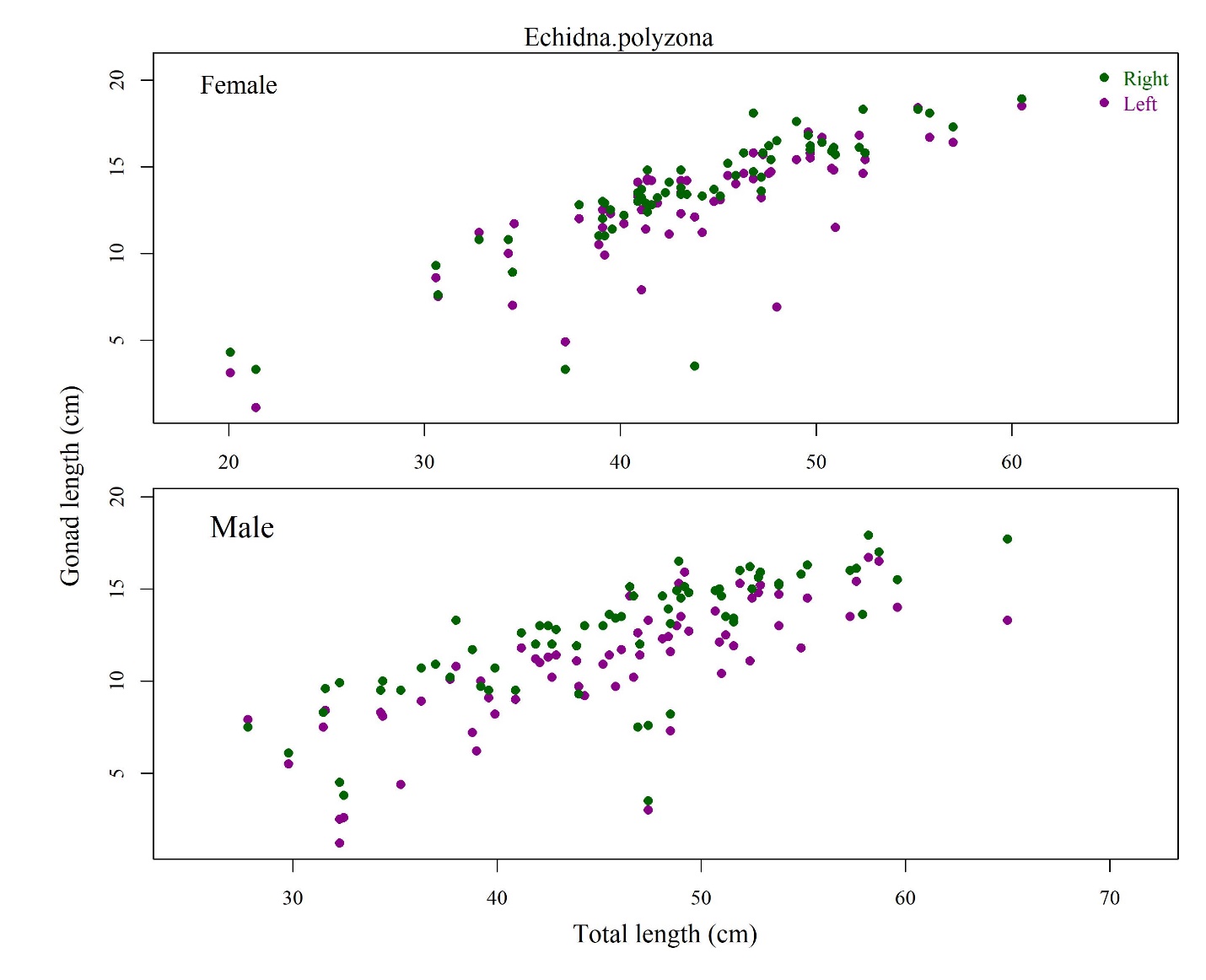


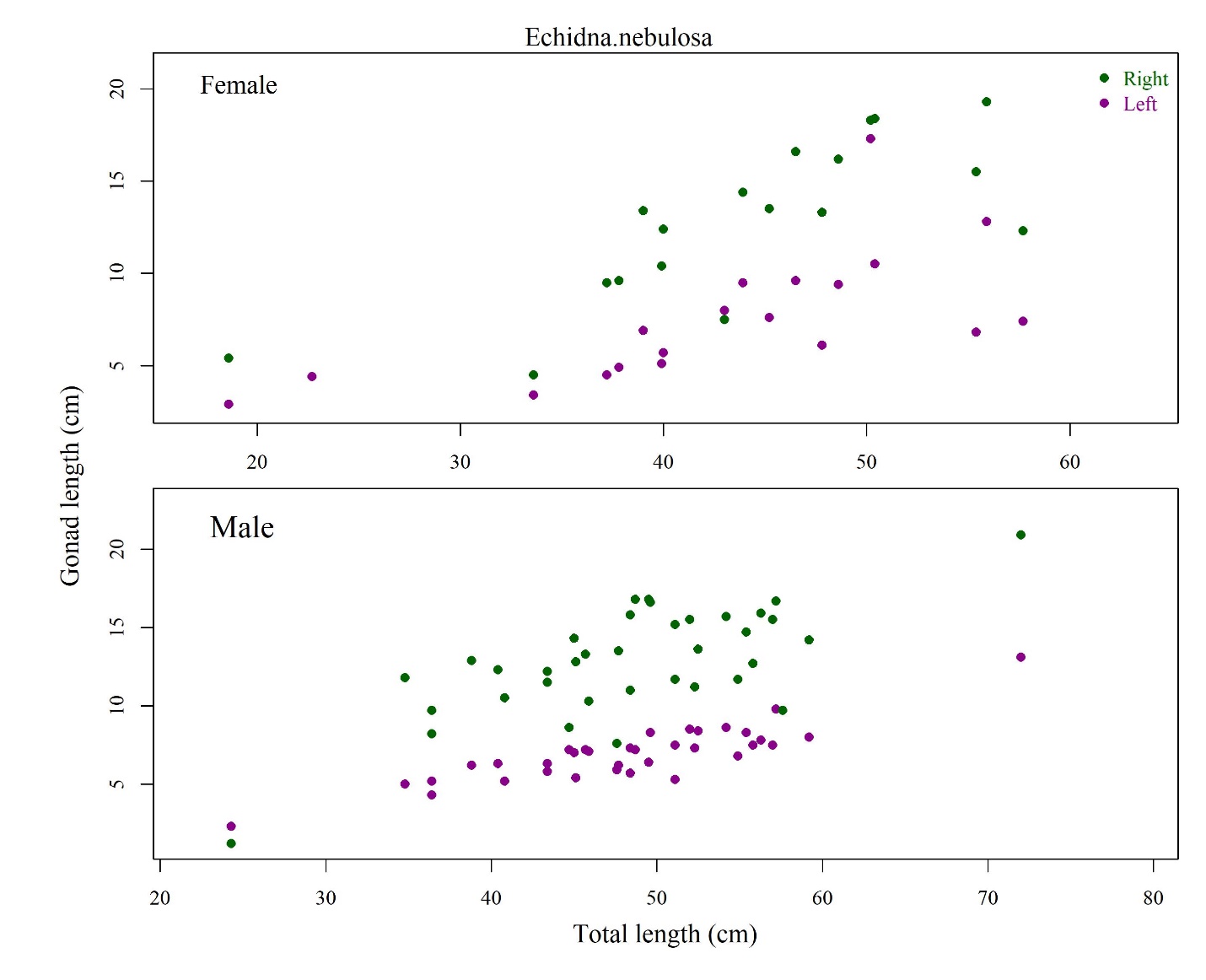


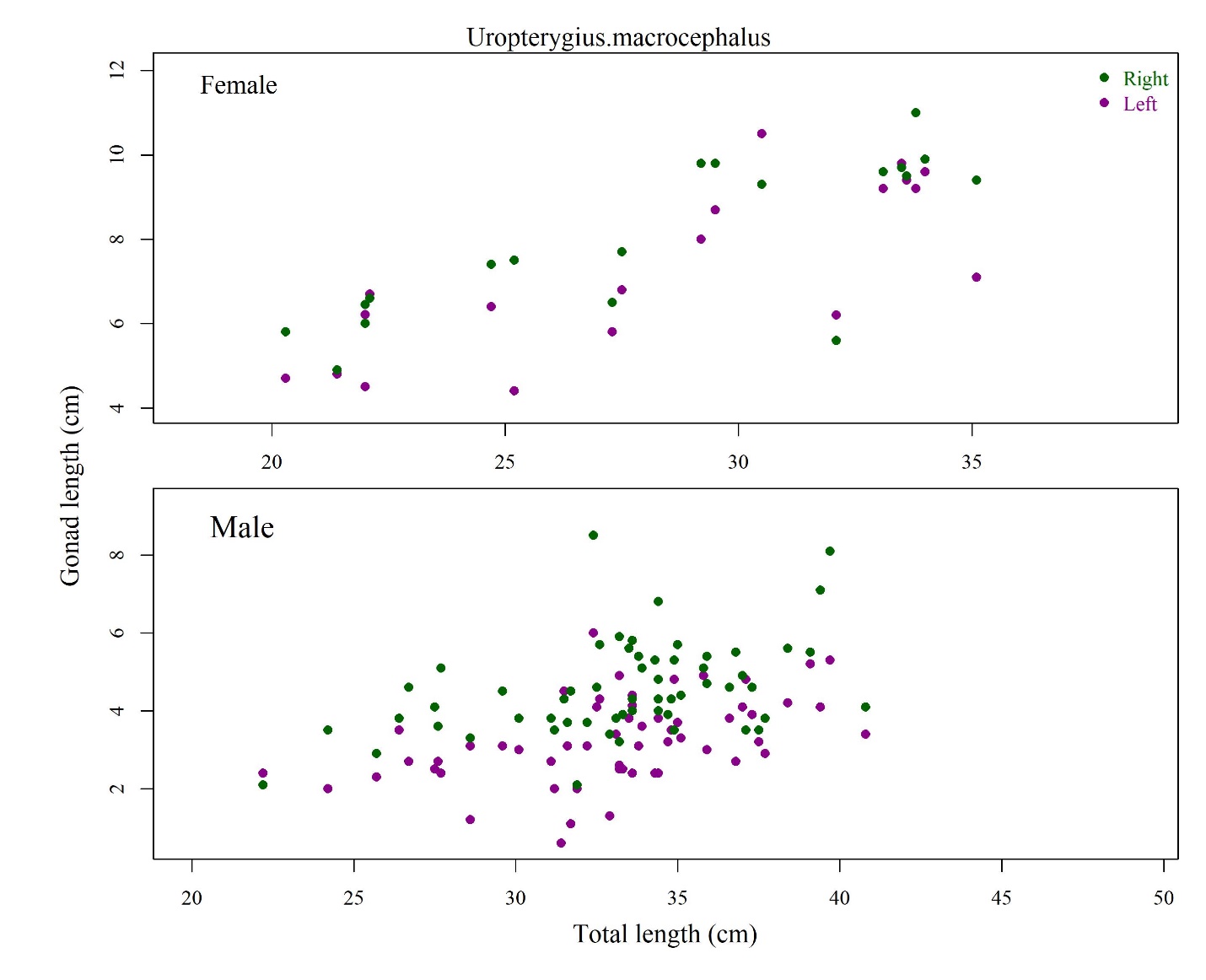


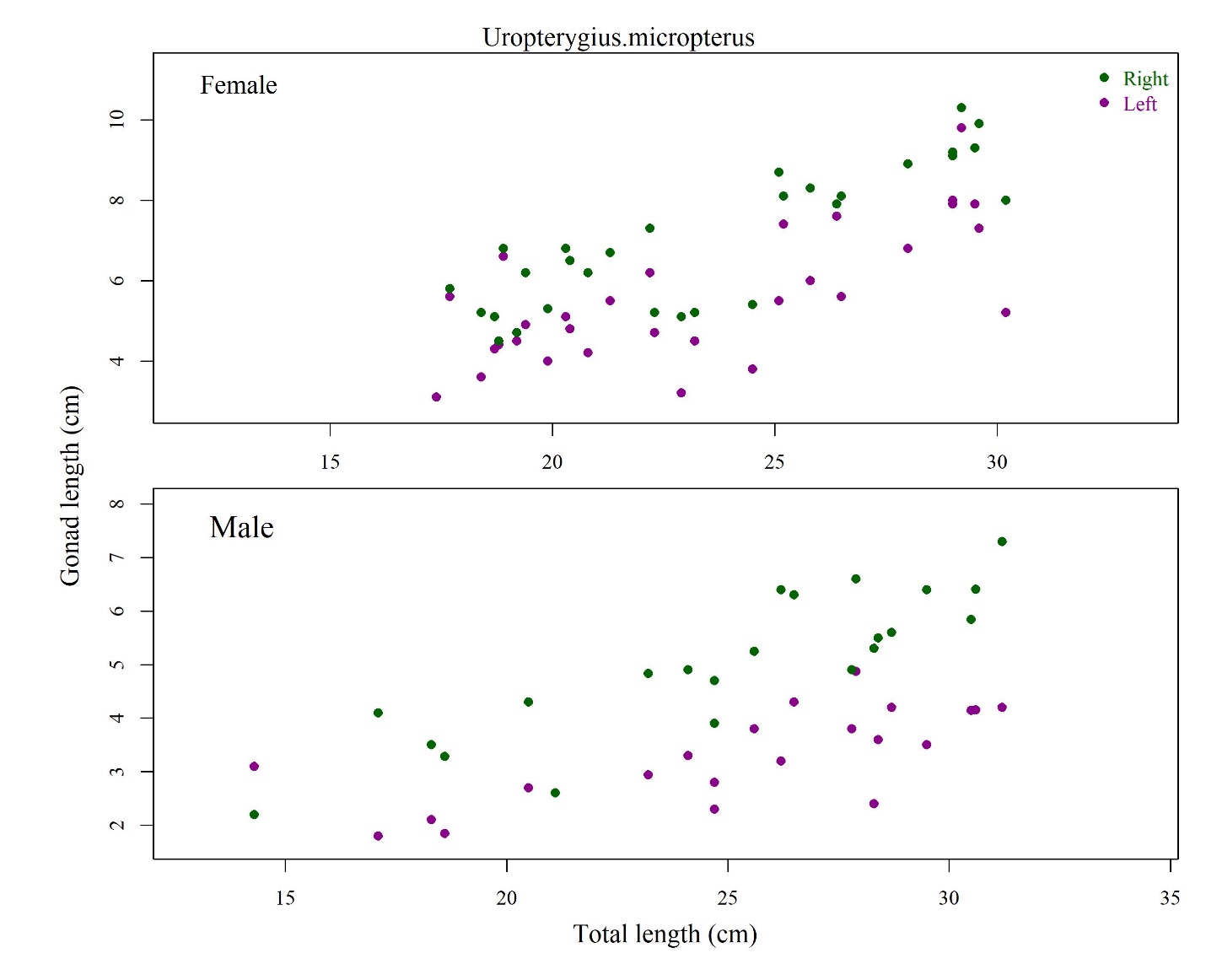


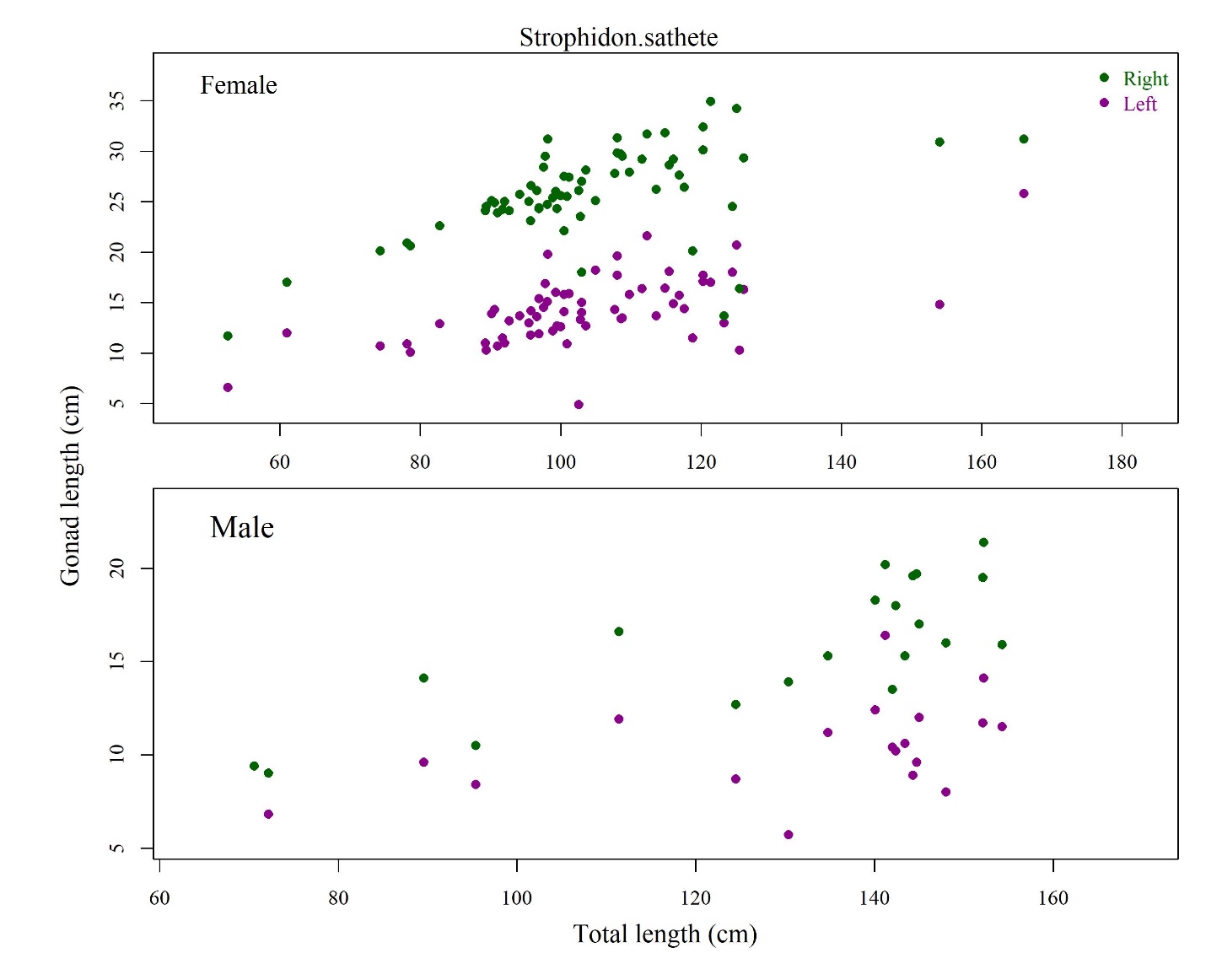


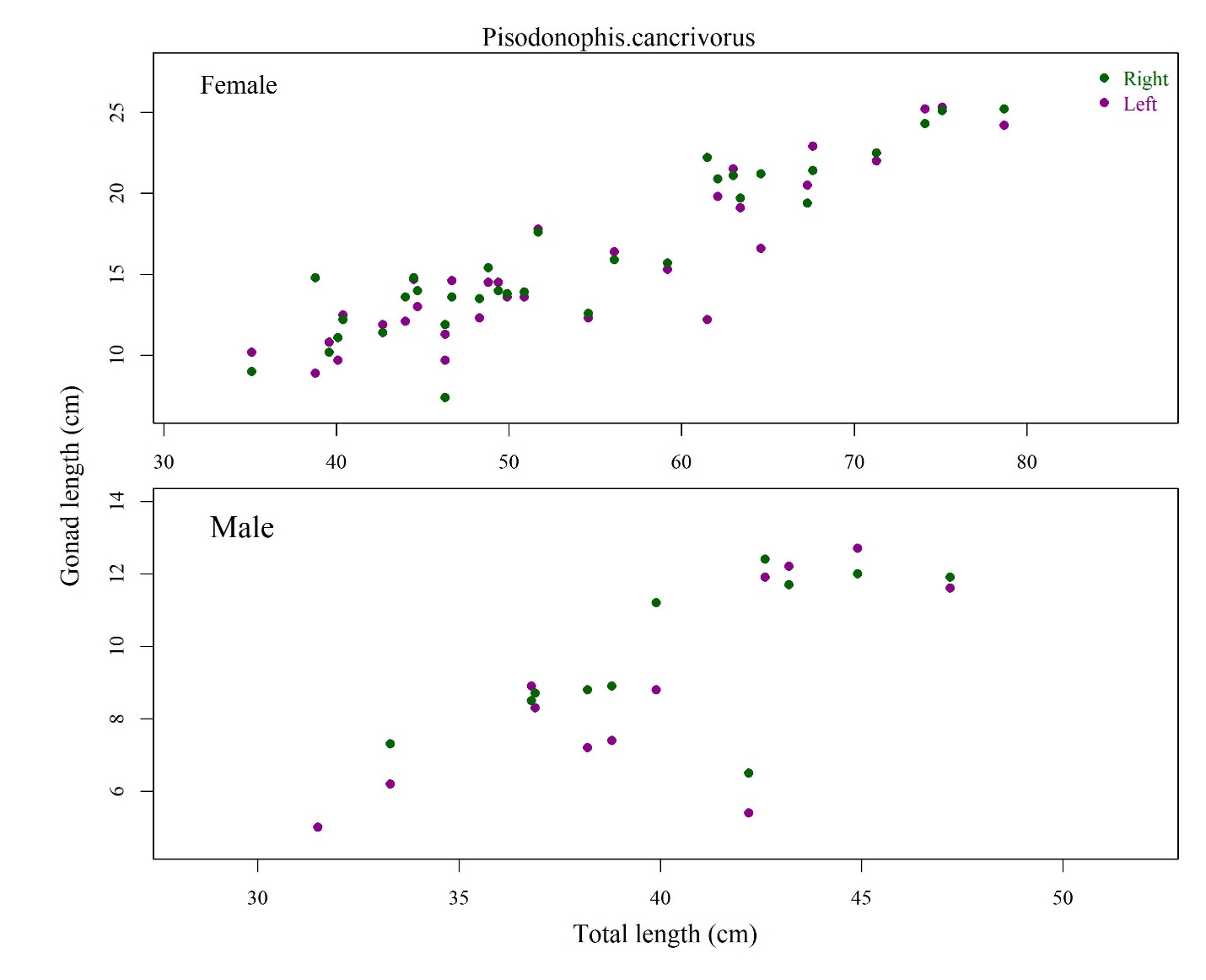


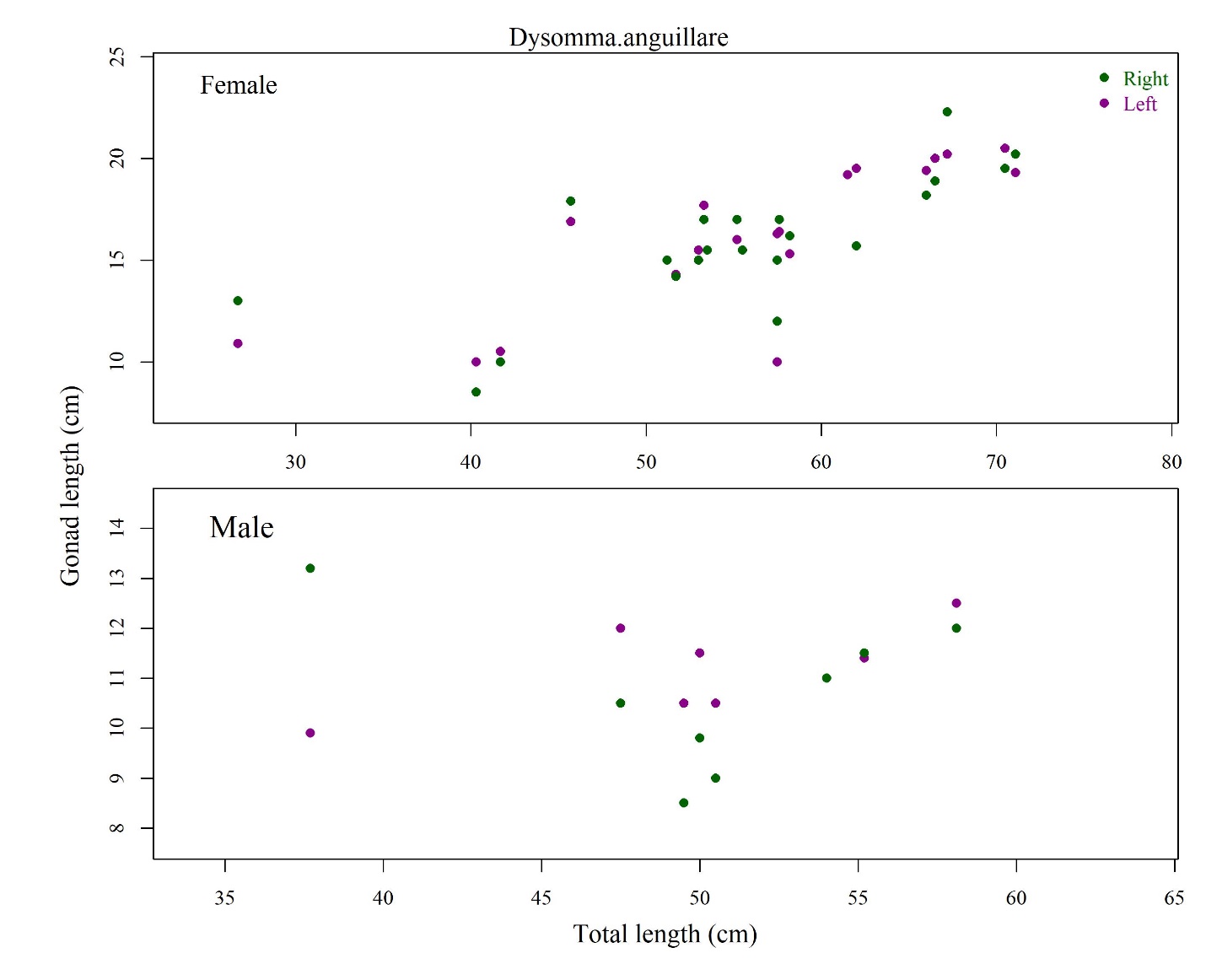


**Supplement material** **6**.

It is common to represent the relationship between the length of body parts (L_part_) to the body size: $L_{part} =A+B\times Size$.

As also shown in Supplement material 3, the gonad lengths of both sides are generally in a linear increasing pattern to the total length for the studied taxa. Then, the lengths of the left and right gonad (GL_L_ and GL_R_) can be represented by the following formulae:

$GL_{L} =A_{L}+B_{L}\times L_{T}$ and $GL_{R} =A_{R}+B_{R}\times L_{T}$,

where GL is the gonad length, L_T_ is the total length, A and B are the intercept and slope of the linear relationship, and subscript L and R indicate the sidedness.

The gonad length difference (GLD, GL_R_ - GL_L_) is a commonly-used measure to quantify the directional asymmetry (refs). The gonad length difference can be represented also by a linear relationship between the gonad length and the total length. Moreover, by using regression the linear effect of the total length can be explicitly evaluated (Refs):

$GLD= GL_{R}- GL_{L} ={(A}_{R}+B_{R}\times TL)-(A_{L}+B_{L}\times TL)$

**=** ${(A}_{R}{-A}_{L})+\left( B_{R}-B_{L} \right)\times TL$

$=A_{D}+B_{D}\times TL$,

where A_D_ = (A_R_ - A_L_) and B_D_ = (B_R_ – B_L_).

The ratio of the gonad length divided by the total length can be represented by;

GL_R_/ L_T_ = ${(A}_{R}+B_{R}\times L_{T})\times L_{T}^{-1}=A_{R}\times L_{T}^{-1}+B_{R}$

$=B_{R}$, if A_R_ = 0

GL_L_/ L_T_ = ${(A}_{L}+B_{L}\times L_{T})\times L_{T}^{-1}=A_{L}\times L_{T}^{-1}+B_{L}$

$=B_{L}$, if A_L_ = 0

Similarly, the ratio of the gonad length from two sides (the right divided by the left) can be represented as:

GL_R_/ GL_L_ = ${(A}_{R}+B_{R}\times L_{T})\times{{(A}_{L}+B_{L}\times L_{T})}^{-1}$

${=B}_{R}\times B_{L}^{-1}$ , if A_R_ = A_L_ = 0

Therefore, the ratio of the gonad length to the total length, and the ratio of the gonad length from each side are not independent of the total length, unless the gonad length is proportional to the total length, i.e., all intercept values are zero for both sides.

**Supplement material 7**. Mitochondrial cytochrome oxidase subunit I (COI) sequence information for GenBank (https://www.ncbi.nlm.nih.gov/genbank/), including the species, accession number, and the region where the specimen is collected.

| **Species** | **Accession number** | **Region** |
| --- | --- | --- |
| *Gymnothorax minor* | KU942739.1 | Taiwan |
| (previously *G. reticularis*) | KU942762.1 | Taiwan |
|  | KU942761.1 | Taiwan |
|  | KU942760.1 | Taiwan |
|  | KU942736.1 | Taiwan |
|  | KU942701.1 | Taiwan |
| *Gymnothorax chilospilus* | KU942754.1 | Taiwan |
|  | KU942753.1 | Taiwan |
|  | KU942752.1 | Taiwan |
| *Gymnothorax eurostus* | KU942757.1 | Taiwan |
|  | KU942756.1 | Taiwan |
| *Gymnothorax shaoi* | KU942759.1 | Taiwan |
|  | KU942766.1 | Taiwan |
|  | KU942767.1 | Taiwan |
| *Gymnothorax fimbriatus* | KU942741.1 | Taiwan |
| *Gymnothorax hepaticus* | MN243507.1 | Red Sea |
|  | MN243506.1 | Red Sea |
|  | MN243505.1 | Red Sea |
| *Gymnothorax flavimarginatus* | KU942758.1 | Taiwan |
| *Gymnothorax kidako* | MH400959.1 | Taiwan |
|  | MF774817.1 | Taiwan |
| *Gymnothorax pseudothyrsoideus* | KU942770.1 | Taiwan |
|  | KU942764.1 | Taiwan |
| *Gymnothorax thyrsoideus* | MK566944.1 | French Polynesian |
|  | MK657992.1 | French Polynesian |
| *Gymnothorax neglectus* | KU942772.1 | Taiwan |
| *Gymnothorax margaritophorus* | KU942750.1 | Taiwan |
| *Gymnothorax pictus* | KU942776.1 | Taiwan |
|  | KU942775.1 | Taiwan |
| *Echidna polyzona* | KU942747.1 | Taiwan |
|  | KU942746.1 | Taiwan |
|  | KU942745.1 | Taiwan |
| *Echidna nebulosa* | KU942744.1 | Taiwan |
|  | KU942743.1 | Taiwan |
|  | KU942742.1 | Taiwan |
| *Uropterygius macrocephalus* | MH400961.1 | Taiwan |
|  | KU942740.1 | Taiwan |
| *Uropterygius micropterus* | MF190360.1 | Taiwan |
|  | MF190361.1 | Taiwan |
|  | MF190362.1 | Taiwan |
|  | MF190363.1 | Taiwan |
|  | MF190364.1 | Taiwan |
| *Strophidon sathete* | KU942773.1 | Taiwan |
|  | KU942735.1 | Taiwan |
| *Pisodonophis cancrivorus* | KU942790.1 | Taiwan |
|  | KU942788.1 | Taiwan |
| *Dysomma anguillare* | KU942780.1 | Taiwan |
|  | KU885613.1 | Taiwan |
|  | KU885612.1 | Taiwan |
